# Sea surface temperature, rather than land mass or geographic distance, may drive genetic differentiation in a species complex of highly dispersive seabirds

**DOI:** 10.1002/ece3.8180

**Published:** 2021-10-04

**Authors:** Lucas Torres, Eric Pante, Jacob González‐Solís, Amélia Viricel, Cécile Ribout, Francis Zino, Will MacKin, Carine Precheur, Julie Tourmetz, Licia Calabrese, Teresa Militão, Laura Zango, Hadoram Shirihai, Vincent Bretagnolle

**Affiliations:** ^1^ Centre d'Etudes Biologiques de Chizé UMR 7372 CNRS ‐ La Rochelle Université Beauvoir sur Niort France; ^2^ Laboratoire LIENSs UMR 7266 CNRS ‐ La Rochelle Université La Rochelle France; ^3^ Department de Biologia Evolutiva, Ecologia i Ciències Ambientals (BEECA) Institut de Recerca de la Biodiversitat (IRBio) Universitat de Barcelona Barcelona Spain; ^4^ Rua Dr Pita Funchal Portugal; ^5^ 3913 Sterling Ridge Ln Durham North Carolina USA; ^6^ Karibiod Lamentin France; ^7^ Société d'Etudes Ornithologiques de La Réunion Saint André France; ^8^ Island Conservation Society Mahé Seychelles; ^9^ Faculty of Business & Sustainable Development Island Biodiversity & Conservation Center University of Seychelles Mahé Seychelles; ^10^ Erga & Dudi Rivis c/o Hadoram Shirihai Kfar Truman Israel

**Keywords:** divergence, mitonuclear discordance, multilocus, philopatry, phylogeography, *Puffinus*

## Abstract

Seabirds, particularly Procellariiformes, are highly mobile organisms with a great capacity for long dispersal, though simultaneously showing high philopatry, two conflicting life‐history traits that may lead to contrasted patterns of genetic population structure. Landmasses were suggested to explain differentiation patterns observed in seabirds, but philopatry, isolation by distance, segregation between breeding and nonbreeding zones, and oceanographic conditions (sea surface temperatures) may also contribute to differentiation patterns. To our knowledge, no study has simultaneously contrasted the multiple factors contributing to the diversification of seabird species, especially in the gray zone of speciation. We conducted a multilocus phylogeographic study on a widespread seabird species complex, the little shearwater complex, showing highly homogeneous morphology, which led to considerable taxonomic debate. We sequenced three mitochondrial and six nuclear markers on all extant populations from the Atlantic (*lherminieri*) and Indian Oceans (*bailloni*), that is, five nominal lineages from 13 populations, along with one population from the eastern Pacific Ocean (representing the *dichrous* lineage). We found sharp differentiation among populations separated by the African continent with both mitochondrial and nuclear markers, while only mitochondrial markers allowed characterizing the five nominal lineages. No differentiation could be detected within these five lineages, questioning the strong level of philopatry showed by these shearwaters. Finally, we propose that Atlantic populations likely originated from the Indian Ocean. Within the Atlantic, a stepping‐stone process accounts for the current distribution. Based on our divergence time estimates, we suggest that the observed pattern of differentiation mostly resulted from historical and current variation in sea surface temperatures.

## Graphical abstract

We conducted a multilocus phylogeographic study on a widespread shearwater and sequenced three mitochondrial and six nuclear markers on all extant populations. We found sharp differentiation among populations separated by the African continent with both markers, while only mitochondrial markers allowed characterizing the five nominal lineages. We propose that Atlantic populations originated from the Indian Ocean. Within the Atlantic, a stepping‐stone process accounts for the current distribution.

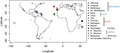

## 1 INTRODUCTION

Population divergence, eventually leading to speciation, is a key process in the study of evolutionary and conservation biology (e.g., Friesen et al., 2007). Though divergence with gene flow has been theorized and observed (reviewed in Pinho & Hey, 2010), the model of allopatric speciation predominates by far in the literature (Stroud & Losos, 2016; The Marie Curie SPECIATION Network, 2012). In this model, a physical barrier to gene flow catalyzes genetic differentiation between populations, through selection and/or genetic drift, eventually followed by other pre‐ or postzygotic barriers (Coyne & Orr, 2004). In practice, however, the mechanisms that impede gene flow and promote differentiation are multifactorial and still difficult to disentangle (Ravinet et al., 2017; The Marie Curie SPECIATION Network, 2012). In particular, geographic barriers alone may not explain the differentiation of populations in highly dispersive species, for example, marine birds (e.g., Genovart et al., 2007), birds of prey (e.g., Doyle et al., 2016), mammals (Moussy et al., 2013), or plants (Sanz et al., 2014). Seabirds are a case in point: Their wide geographic distribution and dispersal ability should theoretically maintain high levels of gene flow, yet many seabirds show surprisingly strong geographic population structure, a pattern that could be attributed, at least partly, to their high degree of philopatry (Friesen et al., 2007).

Present or historic landmasses were identified as the most important barriers to gene flow in seabirds (Friesen, 2015; Friesen et al., 2007) though they cannot explain all differentiation patterns, and other factors such as philopatry, isolation by distance, or segregation between breeding and nonbreeding zones may play a role. However, contrasting these factors simultaneously remains a challenge. The use of sex‐linked markers has proven to be important to understand bird phylogeography, as gene flow is expected to vary between sexes given stronger male philopatry in seabirds and other birds in general (Greenwood, 1980). Sex‐specific patterns of divergence are expected, and indeed, mitonuclear discordance has been detected in seabirds, with more genetic structure in mitochondrial (mt) than in nuclear (nu) DNA (Burg & Croxall, 2001; Deane, 2013; Gangloff et al., 2013; Silva et al., 2015; Welch et al., 2012; but see Pons et al., 2014). Such discordance was suggested to result from incomplete lineage sorting in nuDNA due to a higher effective population size than mtDNA (McKay & Zink, 2010), but other mechanisms were proposed such as adaptive introgression of mtDNA, demographic disparities, and sex‐biased gene flow (reviewed in Toews & Brelsford, 2012).

**FIGURE 1:**
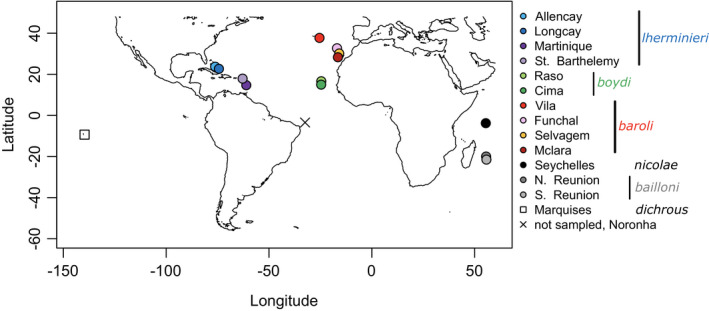
Distribution of the breeding sites for the *Puffinus lherminieri* colonies sampled for this study, with the exception of Fernando de Noronha, which represents the only breeding colony from North Atlantic that was not sampled here. Numbers represent breeding localities that were sampled for this study (color codes identical across figures)

Shearwaters (order Procellariiformes) are long‐lived birds showing slow demographic rates, for example, life expectancy up to or above 15 years (Warham, 1996). They breed in large colonies on remote oceanic islands, are pelagic (González‐Solís et al., 2007), and are highly philopatric (Brooke, 2004). Geographic structuring of populations can be strong (Genovart et al., 2012; Gómez‐Díaz et al., 2009), and the systematics of some taxa is still highly controversial, especially for the little and Audubon's shearwaters *Puffinus assimilis–lherminieri* species complex (Austin et al., 2004). This widespread small‐sized, black‐and‐white shearwater breeds from equatorial to subarctic seas (see map in Figure 1). According to different authors, the number of nominal species in this complex has varied from 1 to 8, and the number of subspecies from 0 up to 26 (review in Austin et al., 2004). Recent studies, based only on the mitochondrial gene *cytb*, indicated marked phylogeographic structure (Austin et al., 2004; Kawakami et al., 2018), with one taxon in the North Atlantic (*lherminieri*), one pantropical taxon (Indian and Pacific Oceans; *bailloni*), and one in the sub‐Antarctic and Australasia (*assimilis*). Using this single marker, three distinct lineages were recognized in the North Atlantic: *lherminieri* in the Caribbean and off Brazil, *baroli* in the Azores, Canaries, and Madeira, and *boydi* in Cape Verde (Figure 1). In the North Atlantic, lineages are characterized by nonoverlapping breeding and nonbreeding distributions at sea (Ramos et al., 2021) and are thus geographically separated. Still, they are morphologically and ecologically highly similar (Precheur et al., 2016), a pattern typical of the first stages of the speciation process, the so‐called gray zone (De Queiroz, 2007). Unsurprisingly, their taxonomic ranking has been hotly debated, for example, *baroli* being considered as belonging to *assimilis* (Shirihai et al., 1995), *lherminieri* (Austin et al., 2004), or a species of its own (Sangster et al., 2005). Lineages belonging to the little and Audubon's shearwater complex are also found in the Indian and Pacific Ocean, with lineages breeding in the Seychelles (*nicolae*), Réunion (*bailloni*) and many islands in the Pacific Ocean (*dichrous*, *gunax*, etc.). Within the *bailloni* lineage, breeding populations are characterized by different breeding phenologies on the northern and southern parts of Réunion (Bretagnolle & Attié, 1996), potentially impacting genetic structuration (Friesen et al., 2007). Indian Ocean birds were alternatively considered as a *P*. *lherminieri* subspecies (Warham, 1990) or subspecies of a *bailloni* pantropical taxon (Austin et al., 2004). The exact taxonomic status of these five lineages (*lherminieri*, *boydi*, *baroli*, *nicolae*, and *bailloni*; Figure 1) is thus largely unresolved. Most recent taxonomies consider either two (Onley & Scofield, 2007) or three different species in the North Atlantic (Flood & Fisher, 2019), and *bailloni* as a single species with two subspecies, *bailloni* and *dichrous* (Onley & Scofield, 2007), or as two distinct species (Howell & Zufelt, 2019).

Here, we consider these five lineages, covering Atlantic and the Indo‐Pacific branches of the *Puffinus assimilis–lherminieri* complex. We sampled birds from all but one known breeding localities in the North Atlantic (four Caribbean breeding sites for *lherminieri*, two main breeding sites in Cape Verde for *boydi*, and one breeding site in Azores, Madeira, Selvagens, and Canary Islands for *baroli*) and in the northern Indian Ocean (northern and southern populations of *bailloni* in Réunion and Seychelles for *nicolae*; Figure 1); we also included three *dichrous* individuals from the Pacific Ocean as to test whether the colonization of Atlantic breeding sites was the result of Indian Ocean bird colonizer or Western Pacific birds passing through the Isthmus of Panama. Only a single *lherminieri* breeding locality was unsampled, on Fernando de Noronha archipelago, off Brazil (Olmos & Silva Silva, 2010; Figure 1). We analyzed three mitochondrial and six nuclear markers to delineate genetic units within the species complex, investigated patterns of genetic differentiation and divergence among populations (i.e., breeding grounds), and discussed implications for their unresolved taxonomy. As conflicting phylogeographic patterns between mitochondrial and nuclear markers (hereon referred to as mitonuclear discordance; Toews & Brelsford, 2012) were systematically found in petrels so far (references above), we inferred population structure among females and males independently to test for sex‐biased dispersal using nuDNA. We expect females to be less structured than males since males are presumably more philopatric (Greenwood, 1980). We then used multispecies coalescent inference and an ABC framework to investigate evolutionary scenarios of breeding site colonization over the last million years. With our inferences, we attempted to disentangle contrasted processes shaping evolutionary history and the contemporary population structure of this complex, such as landmass presence, isolation by distance, oceanographic conditions (sea surface temperature), and past climatic oscillations, as the latter has been shown to influence the present‐day species distributions (e.g., Hewitt, 2004).

## 2 MATERIALS AND METHODS

### 2.1 Sampling, extraction, and PCR amplification of gDNA

A total of 276 birds (all adults, i.e., having already dispersed) spanning the entire known breeding distribution of *Puffinus assimilis–lherminieri* complex in the Atlantic (with the exception of Fernando de Noronha as stated above), as well as three populations of interest for *bailloni* and *nicolae* in the Indian Ocean, were included in our study (metadata in Supplementary Material S1). Three individuals from the Pacific Ocean (taxon *dichrous*, by far the most widespread lineage in the Pacific (Onley & Scofield, 2007; Figure 1) were also included. Individuals were sexed using PCR amplification (with the 2250F and 2781R primers; Fridolfsson & Ellegren, 1999).

Total genomic DNA was extracted from blood samples (except for the Bahamas population, for which samples were derived from toepads collected on dead birds) using NucleoSpin® Tissue XS Kit (Macherey & Nagel, Düren, Germany). Samples were incubated overnight in 4 mg of Proteinase K. Purified genomic DNA was eluted twice in 50 µl of TE buffer preheated at 70℃. DNA concentration was measured using Nanodrop spectrophotometry. Three mitochondrial markers (*cox1*, *cytb*, and the mitochondrial control region, CR) and six nuclear markers (beta‐fibrinogen exon 6 through 8, *βfib*; cold shock domain‐containing E1 intron 5, *csde*; interferon regulatory factor 2 intron 2, *irf2*; PAX‐interacting protein 1 intron 20, *pax*; recombination‐activating protein 1, *rag1*; and tropomyosin 1 alpha exon 7, *tpm*) were targeted (primer sequences, PCR profile, and conditions: Supplementary Material S2). These markers were previously shown to be polymorphic within and among petrel species (Gangloff et al., 2013; Silva et al., 2011).

### 2.2 Quality control of genetic data

While checking chromatograms, we found several cases of double peaks in the sequences of *cox1*, *cytb*, and CR (Supplementary Material S3). Bird blood contains relatively few mitochondria, and it is therefore likely to amplify nuclear copies of mitochondrial markers or “numts” (Sorenson & Quinn, 1998). Such nuclear copies may diverge from the original mitochondrial genes since they are noncoding, which result in double peaks on the chromatograms. To check this scenario and avoid such copies, we digested nuclear DNA with the Exonuclease V (ExoV; NEB‐M0345S) and sequenced again the mitochondrial markers for all individuals showing double peaks for *cox1*, plus 5 individuals showing no double peaks randomly chosen, using a protocol modified from Jayaprakash et al., 2015 (see Supplementary Material S3). Before running analyses, we checked that coding sequences contained no stop codon or indel. Some analyses required phased data (e.g., *BEAST analysis), so the gametic phase of nuclear markers was determined probabilistically using PHASE 2.1 (Stephens et al., 2001) implemented in DNAsp v.5.10.01 (Librado & Rozas, 2009). Additional GenBank sequences (Supplementary Material S1) were aligned to our sequences using MAFFT v 7.187 (Katoh et al., 2002).

### 2.3 Population diversity, differentiation, and divergence

For each population, we calculated haplotype frequencies, interhaplotype distances, haplotype diversity, and nucleotide diversity (*π*), per marker, using DNAsp v.5.10.01 (Librado & Rozas, 2009) and Genetix v4.05.2 (Belkhir et al., 1996). We evaluated signals for departures from neutrality or demographic changes by estimating Tajima's *D* (Tajima, 1989) and Fu's *F*s (Fu, 1997) for each locus, with Arlequin v.3.1 (Excoffier et al., 2005). Differentiation among populations was estimated by performing AMOVAs and calculating pairwise *F*_ST_ and Φ_ST_ and the population average pairwise differences *D*_XY_, using Arlequin (concatenated mitochondrial markers and concatenated nuclear markers). For AMOVAs, samples were stratified into five groups, corresponding to the five nominal lineages (*lherminieri*, *boydi*, and *baroli* in the Atlantic, and *nicolae* and *bailloni* in the Indian Ocean) and populations (i.e., sampling localities; Figure 1) within these groups. The matrix of genetic distances among all pairs of haplotypes was computed using the K2P model of nucleotide substitution for concatenated mitochondrial markers, and TN93 for concatenated nuclear markers, as determined using jModelTest2. We performed a Mantel test to measure the level of correlation among genetic distances (concatenated mitochondrial markers and concatenated nuclear markers) and geographic distances (Smouse et al., 1986). Geographic distance was calculated as the shortest distance between two populations without crossing land. Statistical significance (AMOVAs, pairwise *F*_ST_, and Mantel tests) was estimated using 1000 permutations. To visualize relationships among lineages, we inferred NeighborNet networks using SplitsTree v 4.14.2 (Huson & Bryant, 2006), with different dataset combinations: all markers independently, concatenated mitochondrial markers, concatenated nuclear markers. Finally, we performed a species delimitation analysis with ASAP (Assemble Species by Automatic Partitioning; Puillandre et al., 2020) using the K2P model of nucleotide substitution. ASAP was run on the concatenated *cox1*‐*cytb* sequences (1351 bp, 143 sequences). Including the CR led to excess variation in the dataset and resulted in a spurious delimitation of 89 species. Also, the signal‐poor nuclear sequences were not used for this analysis.

### 2.4 Estimation of sex‐biased dispersal using nuclear markers

To detect sex‐biased dispersal using concatenated nuclear markers, we separated sequences from females and males into two separate datasets, excluding three populations (*lherminieri* from Saint Barthélemy, and *baroli* from Funchal and Selvagem) represented by fewer than five individuals from each sex. Sex‐biased dispersal was tested at both intra‐ and interlineage scales. We calculated average pairwise relatedness for each sex, within each population, using the triadic likelihood estimator (Wang, 2007) implemented in Coancestry (Wang, 2011). To test whether the difference in mean relatedness between males and females of each population was significant, we used the test of difference between sex by bootstrapping samples 1000 times and recalculating difference in means between sexes for each bootstrap. Observed and simulated differences were then compared, and if the observed difference fell outside of the 95% confidence interval, we considered it to be significant.

If females disperse more than males, females sampled from a single population will be a mixture of residents and immigrants. The female sample will therefore deviate from the Hardy–Weinberg equilibrium and show a deficit of heterozygotes (Wahlund effect). *F*_IS_ calculated for the female sample is thus expected to be larger than the male *F*_IS_ (Goudet et al., 2002). We estimated *F*_IS_ separately for females and males for all tested populations, and evaluated its significance using 1000 permutations with Genetix. Conversely, we expect *F*_ST_ (Goudet et al., 2002) to be higher in philopatric males than in females. We calculated *F*_ST_ for each pair of populations within the two datasets, with Arlequin.

### 2.5 Phylogeographic scenarios

Reciprocal monophyly was inferred using gene trees, using all markers as separate partitions. These trees were used to evaluate the degree of divergence among lineages using MrBayes v. 3.2.6 (Ronquist et al., 2012; Supplementary Material S4). To reconstruct the scenario of divergence among the different lineages, we used species trees, inferred using two different methods. We first ran an analysis with *BEAST v 2.2.0 (Bouckaert et al., 2014) using the three mitochondrial markers. We choose to link time‐trees for the three mitochondrial markers, since they are on the same plasmid, where no recombination is expected at the timescale considered in this study. However, the three markers have a different composition and different evolutionary rates, so we did not link the molecular clock and evolution models. We tested the hypothesis of molecular clock with the Clock Test using ML implemented in MEGA v7.0.20 (Kumar et al., 2016). This hypothesis was rejected (*p*‐value <.0005). We therefore used, for each marker, an uncorrelated lognormal relaxed clock model. The clock rate was fixed to 0.00553 ± 0.00115 substitution per site per million year for *cox1* and 0.00631 ± 0.0035 for *cytb* (rates inferred for Procellariiformes by Nabholz et al., 2016). The rate for the control region was estimated by the model as no rate is published for Procellariiformes. Existing rates for other birds could not be used either because they belong to groups that are too distant (e.g., Moas, Baker et al., 2005, or Peafowls, Kimball et al., 1997), and the control region is highly variable among groups (Ruokonen & Kvist, 2002). Published rates for the CR were, however, set in *BEAST as priors and did not affect the results (data not shown). We used for each marker a model consistent with the result of jModelTest2, and a Yule process species tree prior with a continuous population size model. As for the MrBayes analysis, we ran each MCMC chain with 50 * 10^6^ generations, sampled every 1000 generations, the first 25% of generations were discarded as burn‐in. We inspected the stationarity of the chains using Tracer (Rambaut et al., 2018). To test whether the colonization of the Atlantic could result from birds of the Pacific passing through the Isthmus of Panama, individuals from the central Pacific (Marquesas archipelago, taxon *dichrous*) were added to the *BEAST inference. This taxon can be considered as the best representative taxon for the central Pacific, as it is the most widespread and numerous, since *polynesiae* is considered synonym to *dichrous* (Austin et al., 2004), while *bannermani* is best recognized as a species on its own (Kawakami et al., 2018). The taxon *gunax* (from Vanuatu) has never been sequenced, and actually, the location(s) of its breeding colony(ies) is(are) unknown. Three *dichrous cytb* sequences retrieved from GenBank (AY219949‐AY219951) and three individuals from our own collection were used. We ran a second *BEAST analysis by adding all the nuclear markers independently to the three mitochondrial markers, using the same MCMC parameters. Clock rate priors were set to 0.019 substitutions per site per million year for *βfib* and 0.013 substitution per site per million year for *rag1*, since these rates were estimated for birds (Groth & Barrowclough, 1999; Prychitko & Moore, 1997). The clock rate for nuclear markers was estimated by the model as no rate has been produced for petrels. In this latter case, we kept only the individuals for which we had sequences for all markers.

We also used a coalescent‐based ABC approach to explore the best demographic scenario describing the dataset of all mitochondrial markers, and combined mitochondrial and nuclear markers (Supplementary Material S5) using the program DIYABC v. 2.1.0 (Cornuet et al., 2014). ABC methods consist in the simulation of datasets similar to the real dataset in terms of population and marker sizes. First, in the Indian Ocean, we tested whether the three populations emerged simultaneously in a radiation event or in two disjoint events by comparing the posterior probability of these three scenarios, and in the case of the latter, which population was ancestral to the two others and which one of the two remaining populations was ancestral to the other. This hierarchical strategy was applied to each lineage independently, then to the three Atlantic lineages, and finally considering the five lineages together (see Supplementary Material S5 for a description of all tested scenarios). For each possible scenario, 10^6^ pseudo‐observed datasets were simulated, with the same ploidy and number of loci per population as observed in the real dataset. We fixed uniform priors for population sizes, times of size variation and divergence, and mutation and admixture rate priors (see Supplementary Material S4 for details), from which we simulated the datasets. Summary statistics were calculated from the simulated datasets and compared with the same statistics obtained from the real dataset. The Euclidean distance was calculated between the statistics obtained for each normalized simulated dataset and those for the observed dataset (Beaumont et al., 2002). Posterior probability of each scenario was then calculated using a logistic regression on summary statistics produced by the 1% of the simulated datasets closest to the real dataset. To reduce the dimensionality of the data, a linear discriminant analysis was preliminarily applied to the summary statistics (Estoup et al., 2012). The scenario with the highest posterior probability value with a nonoverlapping 95% confidence interval (95% CI) was selected.

## 3 RESULTS

### 3.1 Patterns of genetic diversity, numts, and the presence of a duplicated region

We obtained an average of 192 sequences per marker (length range 307–1323 bp; Table 1). Mitochondrial data exhibited 148 polymorphic sites yielding 150 haplotypes, while nuclear data exhibited a total of 111 variable sites and 150 alleles (see Table 1 for summary of polymorphic sites, haplotypes, and diversities per marker). For mitochondrial markers, the greatest haplotype and nucleotide diversity were observed for the CR (Table 1). For nuclear markers, *βfib* presented the highest number of segregating sites and nucleotide diversity. Mitochondrial markers were more variable than nuclear markers in terms of nucleotide diversity (Table 1).

**TABLE 1:** Summary statistics of polymorphisms for the nine markers used in this study

Marker	*N*	*L*	*S*	*h*/*a*	*hd*	*π*
*cox1*	225 (212, 10, 3)	577	18	12	0.843	0.01301
*cytb*	230 (205, 3, 22)	877	50	48	0.951	0.01394
CR	181	307	80	98	0.993	0.07497
*tpm*	184	427	2	3	–	0.00011
*irf2*	182	542	6	6	–	0.00048
*csde*	223	542	15	13	–	0.0006
*pax*	255 (227, 28)	515	9	10	–	0.00159
*rag1*	162	1323	29	65	–	0.00337
*βfib*	92	1067	50	53	–	0.0083

Note

*N* is the number of sequences obtained (with numbers in parentheses referring to the sequences obtained from this study, obtained from previous studies and downloaded from GenBank); *L* is the length of the sequences in bp; *S* is the number of polymorphic segregating sites; *h*/*a*: *h* is the number of corresponding haplotypes for mitochondrial markers, and *a* is the number of alleles for nuclear markers; *hd* is the haplotype diversity; and *π* is the nucleotide diversity. Due to the presence of ambiguities in the sequence for CR, two haplotype phases were considered here.

None of the coding markers (*cox1*, *cytb*, and *pax*) presented any insertion, deletion, nonsense, or stop codon following translation (see Supplementary Material S1). Double peaks on Sanger chromatograms were, however, detected for each of the three mtDNA markers. While all double peaks at *cox1* were removed by the exonuclease treatment, 60 CR sequences (33%) still showed double peaks at 73 positions, as well as 37 positions for 22 individuals (10%) for *cytb*. Double peaks were not specific to any population or sex and were not linked to the position of the individuals in the sequencing plate (see Supplementary Material S3). Only 12 (5%) individuals showed double peaks both at CR and at *cytb*, so the presence of double peaks seemed unlinked between the two markers. Replicating DNA extractions, PCR, and sequencing confirmed these results, making laboratory contamination unlikely. Contamination in the field was also unlikely since new sampling supplies were used for every sample. Given that only 10% of the *cytb* sequences presented such ambiguities (which may be due to heteroplasmy; Torres et al., 2018), we removed such sequences for further analyses. However, for the CR, since a third of the total sequences were involved, we kept all CR data in further analyses, considering two haplotype phases for MrBayes and *BEAST analyses (although we recognize that these data are in violation of the assumption that sequences to be phased are under Hardy–Weinberg equilibrium; see discussion below).

### 3.2 Population structure and sex‐biased dispersal

Mitochondrial and nuclear results from Fu's *F*s indicated no deviation from neutrality (Supplementary Material S7). However, for Tajima's *D* tests, three localities displayed significant negative Tajima's *D* for all mitochondrial and nuclear markers: South Reunion (Indian Ocean; see map in Figure 1), Saint Barthélemy (W Atlantic), and Raso (E Atlantic). In addition, three localities presented significant negative Tajima's *D* at mitochondrial loci only (Selvagem and Funchal, E Atlantic; South Reunion) and two at nuclear loci only (Vila, E Atlantic; North Reunion). Patterns of population structure at seven out of 13 localities might therefore be influenced by selection and/or recent demographic changes, in addition to neutral processes.

**FIGURE 2:**
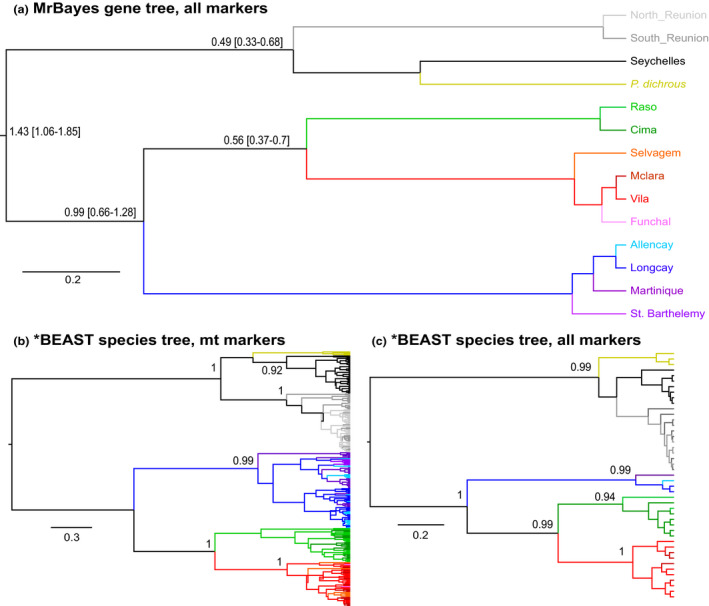
Gene trees and scenario of breeding site colonization. (a) Gene tree obtained by Bayesian inference for all markers, node bars correspond to the 95% confidence interval of the estimated divergence times. The scale corresponds to time before present in Million years (My). (b) Species tree obtained using *BEAST for all mitochondrial markers with dichrous haplotypes in yellow. (c) Species tree obtained using *BEAST for all mitochondrial and nuclear markers with dichrous sequences in yellow. In (b and c), only individuals sequenced for all mitochondrial markers and all markers respectively are shown. Only the posterior values >0.90 are shown

**FIGURE 3:**
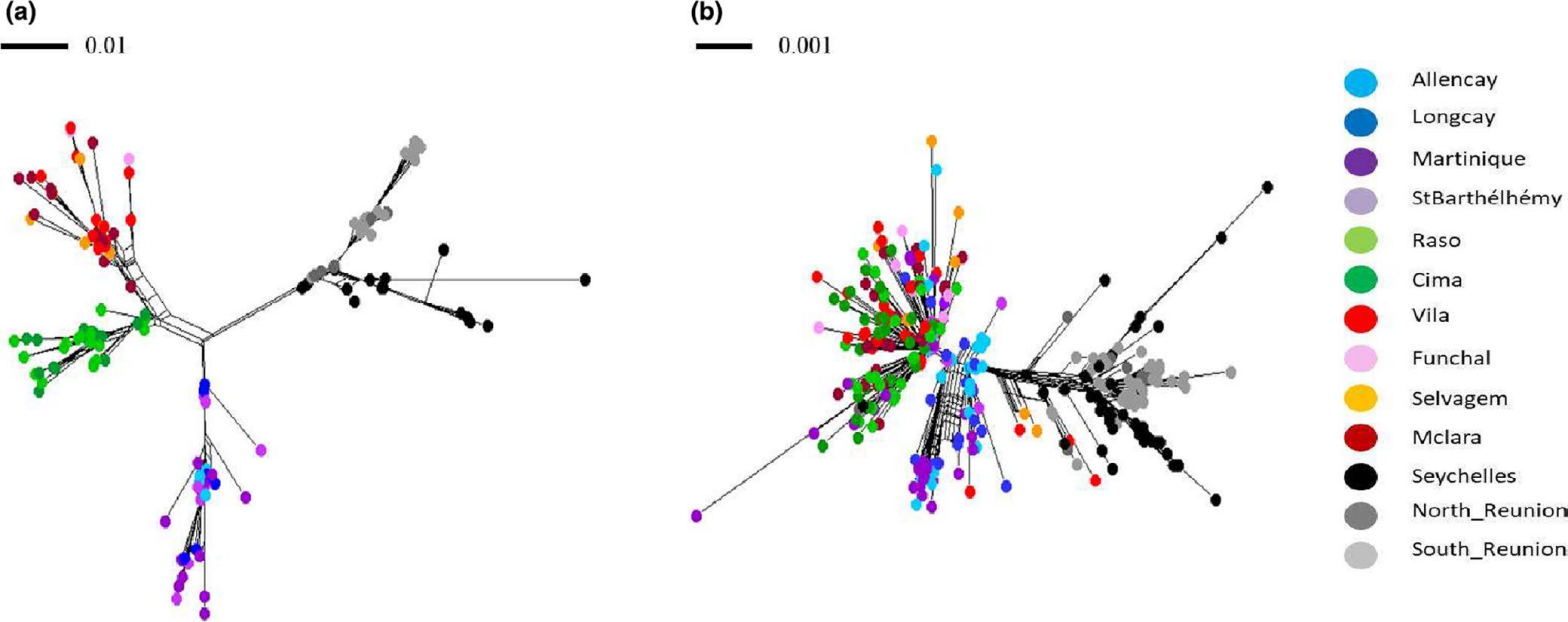
NeighborNet networks obtained using mitochondrial markers (a) and nuclear markers (b). The scale bars indicate the sequence divergence (number of substitutions per site) represented by the length of a branch

Gene trees and phylogenetic inference with *BEAST and MrBayes& revealed a hierarchical structure composed of two well‐supported (posterior probabilities PP ≥ 0.95) reciprocally monophyletic clades corresponding to the two oceans, within which individuals from the five lineages further clustered into monophyletic subclades (Figure 2b,c and S7). All except one of these subclades (East Atlantic *boydi*) were supported in *BEAST (PP ≥ 0.95) using all concatenated markers. For both mtDNA markers, and all markers concatenated, the central Pacific *dichrous* lineage was nested within the *bailloni*/*nicolae* clade, although node supports for *dichrous* position within Indian Ocean clade were weak (PP ranging between 0.27 and 0.73). Assignment to an ocean basin based on nuclear haplotype networks was, however, discordant from the mitochondrial data for 33 individuals, for at least one nuclear locus (Figure 3b and S8): 15 Atlantic individuals fell closely to the Indian nuclear phylogroup, and 18 Indian Ocean individuals clustered within the Atlantic group. All of these 33 individuals showed the mitochondrial signature expected based on their geographic sampling location. Interestingly, a *baroli* individual showed one haplotype phase clustering with the *baroli* phylogroup (mother), while the other haplotype phase (father) clustered with the *nicolae* phylogroup for four nuclear markers (the two remaining could not be assigned to any particular lineages). This individual might be the result of hybridization, although further analyses based on additional markers would be necessary to detect more robustly hybridization among lineages. The ambiguous assignment of the other individuals might be due to introgression or incomplete lineage sorting (see below).

In parallel, we used an AMOVA framework with the five nominal lineages now defined a priori, to examine how genetic variants partitioned among and within these taxonomic units. Most of the genetic variance was due to interlineage differentiation (88.5% and 58.4% for mitochondrial and nuclear markers, respectively). The variance among sampling localities within lineages accounted for 0.5–4.1%, while variance within sampling localities represented 11.0–37.5%. Pairwise *F*_ST_ showed consistently higher values among than within lineages for both marker types, with mostly nonsignificant values within each lineage (Table 2a). Moreover, 24 nuclear *F*_ST_ values were found nonsignificant versus 10 mitochondrial Φ_ST_ values (Table 2a). Population average pairwise differences led to similar results, with high structuration for the five nominal lineages (Supplementary Material S7).

**TABLE 2:**
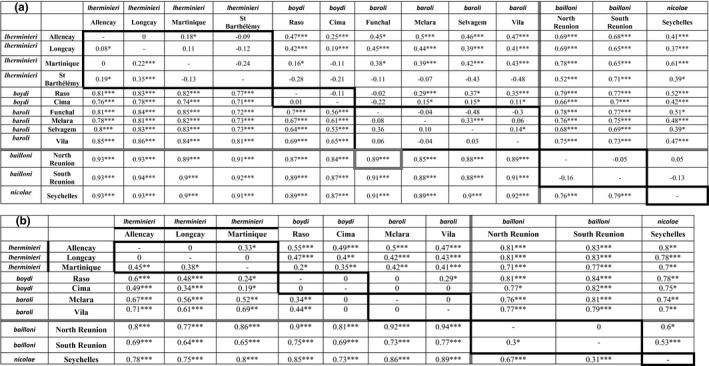
Population differentiation, according to the types of genetic markers and sex

Note

(a) Pairwise Φ_ST_ values for mitochondrial markers (below diagonal) and *F*_ST_ for nuclear markers (above diagonal).

Border indicates the separation between intra and inter lineage comparisons. Triple band indicates the separation between intra‐ and interocean comparisons. *p < .05; ***p < .001.

(b) Pairwise *F*_ST_ for nuclear markers for females (below diagonal) and males (above diagonal).

**FIGURE 4:**
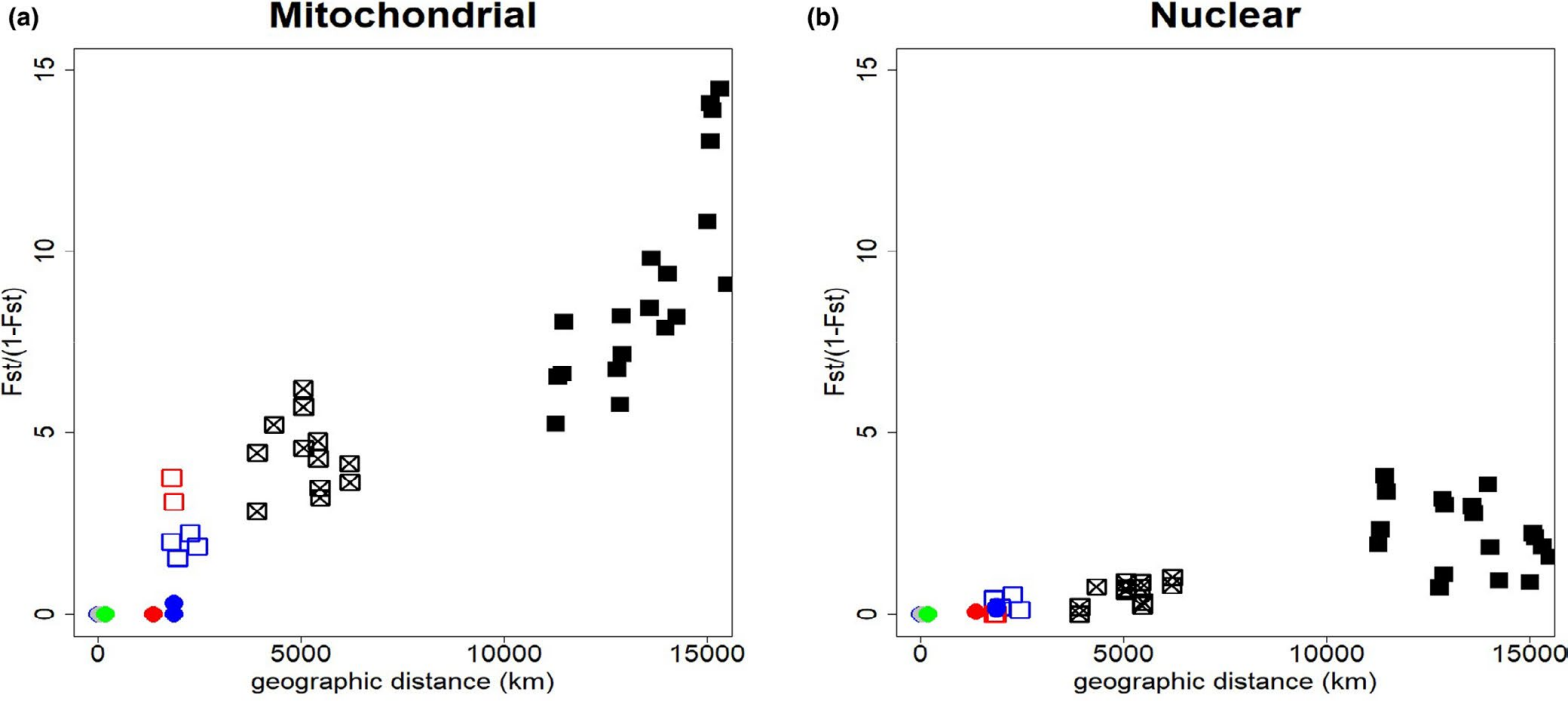
Correlation between genetic and geographic distance. Relationships between genetic (*F*_ST_/(1 − *F*_ST_)) and geographic distances. Genetic distances were calculated for all concatenated mitochondrial markers (a) and all concatenated nuclear markers (b). Geographic distances were calculated as the shortest distances between pairs of populations without crossing land. Black squares: pairs of populations from Atlantic and Indian Oceans. Light squares: pairs of population between *lherminieri* and *baroli*‐*boydi (crossed)*, *boydi* and *baroli (in blue)*, and *bailloni* and *nicolae (in red)*. Colored circles include only pairs of populations belonging to the same lineage

Genetic distance increased clearly with geographic distance (Figure 4a), but Mantel tests were performed only between pairs within the same ocean (given that each oceanic taxon is likely different species). Tests confirmed that genetic and geographic distances were strongly correlated to each other, both for mtDNA and for nuclear markers when analyzing pairs of populations within an oceanic basin (*r *= 0.88 and 0.70, *n *= 45, *p* < .005 for mtDNA and nuDNA, respectively; Figure 4a,b). Between breeding sites and within lineages, isolation by distance could not be reliably tested as the number of populations was too low, but visually it seemed that there was no relation between geographic and genetic distances (Figure 4).

The ASAP species delimitation analysis (*cox1* and *cytb* concatenated) supported four groups, based on the best asap‐score (asap‐score = 5, *p*‐val = 2.3e−1, *W*‐statistic = 3.7e−6, threshold distance = 0.005847). The lineages *herminieri*, *boydi*, and *baroli* were each considered as a separate species, while *bailloni* and *nicolae* were clustered as a single species.

The overall sex ratio was unbiased (120 females, 107 males; Pearson's Chi² with Yates' continuity correction, *p* = .42); 49 individuals could not be sexed successfully and were therefore excluded from sex‐biased dispersal analyses. Indian Ocean populations (*nicolae* and *bailloni*) showed stronger female dispersion as indicated by significantly stronger deficit of heterozygotes and a significantly lower average relatedness in females (Table 3). Conversely, in *baroli*, *F*_IS_ was significantly higher for males and they were less related to each other than females, suggestive of male‐biased dispersal. Finally, an ambiguous pattern was found for both *lherminieri* (males had stronger deficit of heterozygotes and were significantly more related to each other than females) and *boydi* (female *F*_IS_ was significantly higher than male *F*_IS_, though females were more related to each other than for males at one sampling locality). In addition, population structure within lineages, as measured with *F*_ST_, was similar between sexes, but between oceans, a larger range of *F*_ST_ values was observed for males with higher maximum values, suggesting that males were more structured at least for some pairs of populations (e.g., *lherminieri* vs. Indian Ocean lineages; Table 2b). Overall males seemed more structured than females between oceans, suggesting that females disperse farther, but genetic signal for sex‐biased dispersal varied geographically: female‐biased in the Indian Ocean, and male‐biased or inconclusive in the Atlantic Ocean.

**TABLE 3:** Sex‐specific *F*_IS_ and relatedness indices

Lineage	Population	Female sample size	Male sample size	*F*_IS_ Female	*F*_IS_ Male	Observed relatedness female	Observed relatedness male	Observed difference of relatedness	95% CI of inferred difference
*lherminieri*	Allencay	8	10	**0.79 [0.77–0.90]**	**0.35 [0.27–0.43]**	0.45	0.55	−0.10	−0.14: 0.13
*lherminieri*	Longcay	8	12	**0.19 [0.13–0.20]**	**0.60 [0.55–0.69]**	0.62	0.71	−0.09	−0.08: 0.09
*lherminieri*	Bahamas	16	22	**0.63 [0.59–0.67]**	**0.76 [0.75–0.79]**	0.55	0.62	−0.07	−0.07: 0.07
*lherminieri*	Martinique	19	22	0.71 [0.64–0.71]	0.62 [0.59–0.66]	0.70*	0.63*	**0.07**	**−0.04: 0.04**
*boydi*	Raso	11	7	0.53 [0.49–0.58]	0.51 [0.49–0.55]	0.49*	0.38*	**0.11**	**−0.11: 0.10**
*boydi*	Cima	10	9	**0.77 [0.76–0.78]**	**0.66 [0.64–0.73]**	0.56	0.57	−0.01	−0.08: 0.09
*baroli*	Mclara	6	9	**0.36 [0.29–0.39]**	**0.58 [0.55–0.60]**	0.59	0.55	−0.04	−0.08: 0.08
*baroli*	Vila	10	8	**0.48 [0.44–0.54]**	**0.64 [0.63–67]**	0.47	0.45	0.02	−0.09: 0.09
*bailloni*	North Reunion	14	8	**0.77 [0.75–0.82]**	**0.33 [0.23–0.43]**	0.68*	0.78*	**−0.10**	**−0.06: 0.07**
*bailloni*	South Reunion	17	11	**0.58 [0.58–0.66]**	**0.42 [0.34–0.49]**	0.6*	0.72*	**−0.12**	**−0.07: 0.08**
*nicolae*	All populations	14	10	**0.61 [0.59–0.68]**	**0.42 [0.39–0.46]**	0.6*	0.72*	**−0.12**	**−0.09: 0.09**

Note

Mean and confidence interval of *F*_IS_ and relatedness are indicated for each population with a sufficient sample size and for each lineage. The observed difference of mean is considered as significant when not in the confidence interval (indicated in bold and by an asterisk).

### 3.3 Reconstructing scenario of breeding site colonization

**FIGURE 5:**
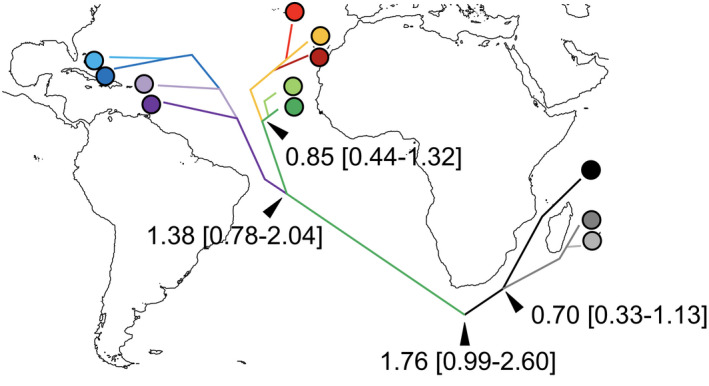
Scenario of colonization inferred based on DIYABC. Branch colors correspond to ancestral populations, dates to mean divergence times of trees in My inferred by *BEAST analyses. Each bifurcation corresponds to a divergence–colonization event. The analysis excludes the population of Funchal, due to low sample size (4 individuals)

A split between ancestral Atlantic and Indian populations (Figures 2 and 5) occurring 1.76 My ago (95% CI range 0.99–2.60) was inferred based on all 9 gene trees (approximately 2.71 My ago (1.17–4.72) using only mitochondrial markers). West and east Atlantic ancestral populations split around 1.38 My ago (0.78–2.04), *baroli* and *boydi* split at approximately 0.85 My ago (0.44–1.32), and *nicolae* and *bailloni* split at 0.70 My ago (0.33–1.13). The *BEAST analysis based on all nine markers showed the same topology, though, with generally lower divergence times and higher confidence intervals (Supplementary Material S8). ABC analyses also supported a similar scenario of ancestral population divergence: Best retained topologies using mtDNA markers and all nine markers suggested, starting from oldest to newest splits, *nicolae* and *boydi* diverged from a common ancestor (Figure 5, Supplementary Material S5). Then, *lherminieri* diverged from *boydi*, and *baroli* diverged from *boydi*. Finally, *bailloni* diverged from *nicolae* (Figure 5, Supplementary Material S4). Our phylogenetic trees placed the Central Pacific taxon *dichrous* within the Indian clade, thus supporting the putative scenario of Atlantic lineages diversifying from Indian Ocean rather than from Pacific ancestors (Figure 2b,c). Within *lherminieri*, ABC analyses suggested a northerly stepping‐stone colonization process, from Martinique to the Bahamas (Supplementary Material S5). Similarly, the most likely scenario of population divergence within *baroli* was colonization from the Canaries to the more northerly Azores.

## 4 DISCUSSION

### 4.1 Mitonuclear discordance and sex‐biased dispersal

At the interlineage scale, we observed more genetic structure at mitochondrial than at nuclear loci. This dissimilarity has been observed for numerous Procellariiformes species (Gangloff et al., 2013; Silva et al., 2015; Welch et al., 2012) and other organisms (Toews & Brelsford, 2012; for a review). One likely explanation is a difference in the pace of molecular evolution between mitochondrial and nuclear markers, with the latter having a slower substitution rate than the former, and the former being more polymorphic at the intraspecific scale (see Brown, 1985), at least in birds (Helm‐Bychowski, 1984; Mindel et al., 1996). However, we did observe high levels of intralineage diversity for some nuclear loci such as *βfib* (see also Gangloff et al., 2013; Silva et al., 2015), and therefore, the difference of structuration cannot be solely attributed to a difference of marker variability. We therefore suspect that incomplete lineage sorting and retention of ancestral polymorphisms at nuclear loci also contribute. Indeed, effective population size of mitochondrial DNA is four times smaller than that of nuclear DNA due to uniparental inheritance and haploid genome. Lineage sorting will therefore be faster in mtDNA than in nuDNA (Funk & Omland, 2003). Incomplete lineage sorting is actually thought to be the main cause of mitonuclear discordance when associated with a pattern of loss of geographic differentiation on nuclear markers (McKay & Zink, 2010; Toews & Brelsford, 2012). Mitonuclear discordance, when diagnostic of mitonuclear genetic incompatibilities, may have a major role in population differentiation (Burton & Barreto, 2012; Hill, 2016, 2017, 2018; Ottenburghs, 2018; Winker, 2021). We also found patterns suggestive of introgression in nuclear markers (e.g., one *baroli* baring both *baroli* and *nicolae* alleles at four nuclear markers). Introgressive hybridization has been documented in shearwaters (Ferrer Obiol et al., 2021; Genovart et al., 2007; Gómez‐Díaz et al., 2009), other Procellariiformes (Brown et al., 2010), and other seabirds (Gay et al., 2009; Morris‐Pocock et al., 2011; Pons et al., 2014). The likelihood of Indian Ocean petrels visiting breeding Atlantic Petrels is also supported by recent tracking of *Pterodroma arminjoniana* breeding on Round Island (Mauritius), which showed that one individual (of mixed ancestry) foraged around South Trinidad Is, off Brazil, and even in the northern Atlantic (Booth Jones et al., 2017), although flight capacities of *Pterodroma* are far higher than *Puffinus*. Introgression can also blur phylogeographic signals by mixing alleles from distinct populations and is considered as the second main cause of mitonuclear discordance (McKay & Zink, 2010). Incomplete lineage sorting and introgression are, however, difficult to distinguish, and additional unlinked markers would be required to disentangle these phenomena. Finally, as the mitochondrial markers represent only the female evolutionary history, sex‐biased dispersal favoring females may alternatively explain why the population structure inferred based on nuclear markers conflicts with female‐inherited mtDNA markers (see Petit & Excoffier, 2009). These authors suggested that the markers associated with the most dispersing sex should better delimitate species, as they will show stronger intraspecific gene flow from colonizing lineages, reducing the effects of genetic drift and lowering the probability of fixating introgressed alleles. We found that dispersal was indeed stronger in females in some populations, particularly in the larger and the putatively ancestral lineage, *nicolae*. Sex‐biased dispersal was, however, more uncertain for *lherminieri* and *boydi*, while for *baroli*, dispersal was inferred to be male‐biased. The sample size for *baroli* was theoretically large enough to robustly detect a bias in *F*_ST_, *F*_Is_, and relatedness (Goudet et al., 2002). Sex‐biased dispersal may therefore have further contributed to the observed mitonuclear discordance, but only in some lineages.

### 4.2 Sequencing artifacts due to mtDNA duplication and uncertainties about molecular clock rates

We found a duplicated region comprising two copies of the CR in our study (Torres et al., 2018), yielding the presence of double peaks on chromatograms. The mitochondrial genome of several Procellariiformes presents tandem repeats (Abbott et al., 2005; Lounsberry et al., 2015). By treating gDNA with an exonuclease (which effectively removed all linear DNA), we did not observe triple or quadruple peaks on chromatograms and thus hypothesized that only two copies of the CR sequences were amplified by PCR. Therefore, we considered two haplotype phases for all CR sequences, assuming that removing all CR sequences would have led to a loss of information and of statistical power in the analyses. To check the robustness of this approach, however, we replicated all our analyses using two other data subsets: one in which all ambiguous CR sequences were removed, and another one from which all CR sequences were removed. Removing all CR sequences led to a strong loss of information, an increase in the estimated differentiation, and a decrease in the estimated divergence times (Supplementary Material S6). Removing only the ambiguous sequences led to estimations close to the estimations of the complete dataset. This suggests that the noise caused by the multiple copies of CR was swamped by the signal contained in that marker and so our analyses using all the individuals are not significantly biased by these sequences.

Another major issue concerns the choice of a molecular clock rate for dating the splitting events. We found a clear hierarchical structuration of populations in the Atlantic and Indo‐Pacific Oceans, which diverged around 1.76–2.71 My ago either using all markers or only mtDNA markers, respectively. In contrast, using the same taxa (but fewer specimens and only *cytb*), Austin et al. (2004) dated this split at 3.2–3.8 My, and rather suggested that the closure of the Isthmus of Panama erected a barrier to gene flow between Indo‐Pacific and Atlantic populations. This difference in divergence times might be due to taxon sampling, gene sampling, or the calibration of the molecular clock (0.631%/My here, vs. 0.9%/My in Austin et al). Actually, using the same value as Austin et al, and only sequences from *cytb*, we found a divergence time of 2.2–3.8 My, thus very close to Austin et al. Many substitution rates for both Mt DNA markers were proposed for petrels, ranging from 0.19%/My (Pacheco et al., 2011) to 1.544%/My (Pereira & Baker, 2006) for *cox1*, and from 0.18%/My (Pacheco et al., 2011) to 0.88–0.92%/My (Nunn & Stanley, 1998; the latter rate was used by Austin et al), 1.022%/My (Pereira & Baker, 2006), and 1.89%/My (Weir & Schluter, 2008) for *cytb*. Using the lowest substitution rates (Pacheco et al., 2011) resulted in a divergence time between Atlantic and Indo‐Pacific lineages around 12.8 My ago (Supplementary Material S10), an unlikely value given the presence of an ocean between the Americas before the Isthmus of Panama erected, despite the fact that *Puffinus* is dating back to the Oligocene (Henderson & Gill, 2010). Here, we used the substitution rates that were estimated in Nabholz et al. (2016), as the most recent review. There are two further arguments against Austin et al. (2004) scenario: First, the closure of the Isthmus of Panama has been actually dated at 2.8 My ago (O'Dea et al., 2016), thus later than Austin et al's scenario; and second, we found that the pacific taxon, *dichrous*, was not ancestral to the Atlantic taxon, but branched within the Indian Ocean clade.

### 4.3 Inferring key drivers of diversification in the small shearwaters

Our inferred date for the split between Indian and Atlantic lineages suggests that gene exchanges between Indian and Atlantic birds were possible even after the closure of the Isthmus of Panama (2.8 Mya; O'Dea et al., 2016). Since African continent is an insurmountable barrier for Procellariiformes (Silva et al., 2015), we suggest that this happened through individuals flying off South Africa. These shearwaters are tropical or subtropical species (at least currently): Off South Africa, until 1 My ago, sea surface temperatures (SST) were approximately 2℃ higher than today (Bell et al., 2015), suggesting that migration between Atlantic and Indian Oceans may have remained possible. Since this latter period, a strong decrease in SST occurred in both oceans (Bell et al., 2015), and gene flow between Indian and Atlantic Oceans may thus have ceased as the Indo/Atlantic colonization corridor implied traversing colder waters. This agrees with our estimated time of divergence (1.76 My ago). Once the Atlantic birds were isolated from Indo‐Pacific populations, differentiation started to occur among Atlantic lineages 1.38–1.90 My ago (respectively for the 9 markers or only the mtDNA markers). This period also corresponds to a further decrease in the SST in the Atlantic (Bell et al., 2015), a southward shift of the subtropical front and warmer waters in the Southern Ocean (Maiorano et al., 2009), and sea ice development in the North extending southward from the Arctic to the current Great lakes in the United States (Webb & Bartlein, 1992). Cold temperatures, preventing the colonization of potential northern breeding sites such as the Azores, may have promoted the colonization of shearwaters over the eastern and western sides of the Atlantic (Figure 5). Divergence of *baroli* from *boydi* would have occurred around 0.85–1.26 My ago, a period that corresponds to a stabilization of the SST at the current level in the North Atlantic. The ice melt may then have allowed northward colonization on both sides of the Atlantic, from Cape Verde to the Canaries, Madeira, and the Azores, and from the lesser Antilles to the Bahamas and Bermuda. Similar timing of divergence has been suggested between *Calonectris edwardsii* (Cape Verde) and *C*. *diomedea* (North Atlantic and Mediterranean) 0.7–0.9 My ago (Gómez‐Díaz et al., 2006) and *Puffinus olsoni* (Canaries) and *Puffinus puffinus* (North Atlantic) 0.2–1.0 My (Ramirez et al., 2010). In the Indian Ocean, a reversed pattern of southward colonization occurred, shearwaters colonizing from Seychelles (partly continental in origin, Seychelles separated from India about 64 Mya; Plummer & Belle, 1995) to Réunion 0.70–1.01 My ago, precisely at the time strong volcanic activity ended on Réunion (Gillot & Nativel, 1989). Mauritius was probably colonized long before (Mauritius age: 8 My; McDougall & Chamalaun, 1969), then Rodrigues and Réunion (both about the same age, 2 My; McDougall, 1971), but shearwaters are now extinct on Mauritius and Rodrigues, following human colonization since the 17th century, so no sample is available. The southward movement in Indian Ocean was likely related to availability of volcanic islands that eventually emerged in a southward direction.

Dispersal limitation has been suggested as a speciation mechanism in seabirds. However, isolation by distance within each lineage was not detected here, since Mantel tests showed a strong correlation between geographic and genetic distances only among lineages, that is, at large scale. Therefore, distance alone could not be a factor of population divergence at this smaller scale. Rather, we argue that sea temperature could be the most important factor of divergence in our case, even at this scale. Seabirds indeed depend on both sea and the islands where they breed; thus, SST has strong impacts on their phenology, breeding, survival, and abundance (Sydeman et al., 2012). Even foraging ecology, which strongly depends on SST, may be an important process shaping divergence among lineages, as segregation of foraging areas among populations is an important factor of differentiation among seabirds (Friesen, 2015; Friesen et al., 2007), shearwaters (Genovart et al., 2007; Gómez‐Díaz et al., 2006), petrels (Gangloff et al., 2013; Welch et al., 2012), storm‐petrels (Deane, 2013; Smith et al., 2007), and albatrosses (Alderman et al., 2005; Burg & Croxall, 2001). Assessment of nonbreeding and breeding distributions at sea of the little shearwaters complex revealed that all three Atlantic taxa show rather separated foraging and wintering areas (Ramos et al., 2021), and further suggest that *boydi* rather than *lherminieri* was ancestral in the North Atlantic. Indeed, *boydi* is more flexible in its foraging ecological niche, suggesting ancestral behavior (Zajková et al., 2017; see map in Ramos et al., 2021). In addition, over the last My, SST oscillations gradually increased in amplitude with up to five degree difference in range (Bell et al., 2015; Herbert et al., 2011), influencing marine productivity (Martínez‐Garcia et al., 2009), prey species diversity (Yasuhara & Cronin, 2008), atmospheric circulation (Chang et al., 2000), and sea level (e.g., in Atlantic Nascimento et al., 2011; Zazo et al., 2010). It is likely that these oscillations also contributed to divergence in the North Atlantic, and we suggest that significant Tajima's *D* tests found in almost half of the populations studied represent traces of past bottlenecks and population expansions as it has already been shown in other taxa (Ramakrishnan et al., 2005; Weber et al., 2004; Zhu et al., 2006). Small black‐and‐white shearwaters have shown a very recent radiation speciation event, with not less than 13 species radiating in just 1.46 million years since *P*. *puffinus*, *P*. *assimilis*, and *P*. *newelli* clades are either embedded in *lherminieri* or *bailloni* clades. All these species are rather coastal shearwaters (compared with more pelagic species such as the larger shearwaters), and such high rate or speciation may be the result of the high climatic oscillation that occurred over the last 2 million years, which may have favored high rates of colonization and extinction on coastal islands.

Our study reveals, in a marine taxon, high levels of genetic differentiation, both between and within oceans, governed mainly by oceanographic parameters, SST in particular. Other marine organisms, however, may not show high interocean structure (e.g., pelagic fishes Díaz‐Jaimes et al., 2010; Ely et al., 2005), nor intraocean structure (Nomura et al., 2014; Taguchi et al., 2015). But gene flow among marine mammals is influenced by SST, driving structuration between ocean basins and among breeding areas (Alexander et al., 2016; Fontaine et al., 2014; Jackson et al., 2014; Richard et al., 2018; Viricel & Rosel, 2014), as do sea turtles (Dutton et al., 1999). Finally, organisms with a pelagic larval phase show globally low structuration (Kelly & Palumbi, 2010), but when detected, structuration is often linked to sea temperature (Benestan et al., 2015; Teske et al., 2005, 2018). Therefore, SST appears as a generic driver of diversification in many marine organisms, not only seabirds, though patterns of structuration are generally weaker (Bowen et al., 2016). We suggest that the reason for this discrepancy lies in the fact that seabirds are central place foragers; that is, they still depend on terrestrial habitats for breeding, the latter being impacted for instance by glaciations. They are therefore highly sensitive to any latitudinal change of SST in comparison with island distribution, which acts as a constraint since an optimal area in regard to SST may lack any island for breeding. We may thus expect marine organisms with low dispersal abilities to show patterns of structuration and divergence similar to the patterns found on shearwaters. Interestingly, timing of population divergence between Atlantic and Indian Ocean lineages and within the Atlantic in the seahorse *Hippocampus* “*kuda* complex” (Floeter et al., 2007) fits to our estimates. This species has no planktonic larval duration (Lourie et al., 2005), and long dispersal events are considered rare and implying a few individuals (Teske et al., 2005). Moreover, Cape Agulhas is known to be a phylogeographic break among several coastal species, due to the difference in currents and sea temperatures between the two oceans (review in Teske et al., 2011).

In this small shearwater complex, geographic barriers and/or isolation by distance may have been a major driver of differentiation at large scale (typically, between oceans), while SST has been a more important driver at smaller scale (within oceans). However, since these seabirds depend on the geographic distribution of their breeding islands and because they are relatively “poor” flyers, island distribution becomes a major constraint resulting in the present geographic structure, promoting local adaptation to small‐scale ecological constraints and reducing gene flow. Strict marine organisms can disperse far more or alternatively are unconstrained by island distribution, and thus show much less geographic structure within taxa. The terrestrial organisms tend to disperse far less, and isolation by distance tends to be a main driver of population differentiation (Meirmans, 2012; Vekemans & Hardy, 2004). Indeed, terrestrial organisms, such as lizards or birds in Macaronesia (Almalki et al., 2017; Brehm et al., 2003), geckos in Cape Verde (Arnold et al., 2008), or birds in America (Patel et al., 2011), have revealed strong splits between islands with no shared haplotypes for the same mitochondrial markers. Therefore, petrels and shearwaters present an interesting case study where diversification processes rely more (or at least equally) on ecological factors, in particular sea surface temperature, rather than distance or continental barriers, in contrast to either “true” marine organisms or terrestrial organisms.

## CONFLICT OF INTEREST

The authors declare no conflict of interest.

## AUTHOR CONTRIBUTIONS

**Lucas Torres:** Conceptualization (equal); data curation (equal); formal analysis (equal); methodology (equal); writing‐original draft (equal); writing‐review & editing (equal). **Eric Pante:** Conceptualization (equal); data curation (equal); formal analysis (equal); methodology (equal); project administration (equal); supervision (equal); validation (equal); writing‐original draft (equal). **Jacob González‐Solís:** Resources (equal); writing‐original draft (equal). **Amélia Viricel:** Formal analysis (equal); writing‐original draft (equal). **Cécile Ribout:** Methodology (equal). **Francis Zino:** Resources (equal); writing‐original draft (equal). **Will MacKin:** Resources (equal); writing‐original draft (equal). **Carine Precheur:** Resources (equal). **Julie Tourmetz:** Resources (equal). **Licia Calabrese:** Resources (equal). **Teresa Militão:** Resources (equal). **Laura Zango:** Resources (equal). **Hadoram Shirihai:** Resources (equal); writing‐original draft (equal). **Vincent Bretagnolle:** Conceptualization (equal); funding acquisition (lead); supervision (equal); writing‐original draft (equal); writing‐review & editing (equal).

## Supporting information

Supplementary Material

## Data Availability

The DNA sequences are submitted to GenBank with accession nos. 42970‐43663.
