# Supplementary material for: Sea surface temperature, rather than land mass or geographic distance, may drive genetic differentiation in a species complex of highly dispersive seabirds
Source: Ecol Evol. 2021 Oct 4;11(21):14960–76. doi: 10.1002/ece3.8180 (PMC8571584; doi:10.1002/ece3.8180)
Supplement: Supplementary file 1 — Supplementary Material [file ECE3-11-14960-s001.docx]

Supplementary Material 1: List of sequences used in this study.

a.

1. Summary table: sample size per lineage, colony, and sex. b. Additional sequences obtained from Genbank

| Lineage | Colony | Sample size | Number of females | Number of males | Genbank accession numbers |
| --- | --- | --- | --- | --- | --- |
| *lherminhieri* | Allencay | 19 | 8 | 10 | XXXX |
| *lherminhieri* | Longcay | 20 | 8 | 12 | XXXX |
| *lherminhieri* | Martinique | 44 | 19 | 22 | XXXX |
| *lherminhieri* | StBathélémy | 8 | NA | NA | XXXX |
| *boydi* | Cima | 18 | 10 | 9 | XXXX |
| *boydi* | Raso | 18 | 11 | 7 | XXXX |
| *baroli* | Funchal | 4 | NA | NA | XXXX |
| *baroli* | Mclara | 15 | 6 | 9 | XXXX |
| *baroli* | Selvagem | 10 | 3 | 1 | XXXX |
| *baroli* | Vila | 19 | 10 | 8 | XXXX |
| *bailloni* | North Réunion | 28 | 14 | 8 | XXXX |
| *bailloni* | South Réunion | 32 | 17 | 11 | XXXX |
| *nicolae* | Seychelles | 41 | 14 | 10 | XXXX |
| *dichrous* | Marquises | 3 | NA | NA | XXXX |
| *pacificus* | Réunion | 1 | NA | NA | XXXX |

| Genus | species | Marker | Genbank accession | Reference | Voucher |
| --- | --- | --- | --- | --- | --- |
| *Puffinus* | *lherminieri* | *cox1* | DQ434015 | Kerr et al. 2007 | USNM 620720 |
| *Puffinus* | *lherminieri* | *cox1* | JQ176049 | Schindel et al. 2011 | USNM:Birds:607634 |
| *Puffinus* | *lherminieri* | *cox1* | JQ176050 | Schindel et al. 2011 | USNM:Birds:607633 |
| *Puffinus* | *lherminieri* | *cytb* | AF076085 | Nunn and Stanley 1998 |  |
| *Puffinus* | *baroli* | *cytb* | AY219935 | Austin et al. 2004 | tissue sample Pabr93 |
| *Puffinus* | *baroli* | *cytb* | AY219936 | Austin et al. 2004 | tissue sample Pabr91 |
| *Puffinus* | *boydi* | *cytb* | AY219937 | Austin et al. 2004 | museum skin BMNH 1936.2.21.87 |
| *Puffinus* | *lherminieri* | *cytb* | AY219940 | Austin et al. 2004 | museum skin BMNH 1913.12.26.75 |
| *Puffinus* | *lherminieri* | *cytb* | AY219941 | Austin et al. 2004 | museum skin BMNH 1932.4.13.1 |
| *Puffinus* | *lherminieri* | *cytb* | AY219942 | Austin et al. 2004 | museum specimen MZUSP 75186 |
| *Puffinus* | *lherminieri* | *cytb* | AY219943 | Austin et al. 2004 | museum tissue sample LSM B20918 |
| *Puffinus* | *lherminieri* | *cytb* | AY219944 | Austin et al. 2004 | tissue sample Pllh_EP7 |
| *Puffinus* | *lherminieri* | *cytb* | AY219945 | Austin et al. 2004 | tissue sample Pllh_EP8 |
| *Puffinus* | *loyemilleri* | *cytb* | AY219946 | Austin et al. 2004 |  |
| *Puffinus* | *lherminieri* | *cytb* | AY219947 | Austin et al. 2004 |  |
| *Puffinus* | *lherminieri* | *cytb* | AY219948 | Austin et al. 2004 |  |
| *Puffinus* | *nicolae* | *cytb* | AY219956 | Austin et al. 2004 |  |
| *Puffinus* | *nicolae* | *cytb* | AY219957 | Austin et al. 2004 |  |
| *Puffinus* | *nicolae* | *cytb* | AY219960 | Austin et al. 2004 |  |
| *Puffinus* | *bailloni* | *cytb* | AY219963 | Austin et al. 2004 |  |
| *Puffinus* | *bailloni* | *cytb* | AY219964 | Austin et al. 2004 | tissue sample Plba_VB |
| *Puffinus* | *boydi* | *cytb* | L43024 | Austin 1996 |  |
| *Puffinus* | *boydi* | *cytb* | L43025 | Austin 1996 |  |
| *Puffinus* | *lherminieri* | *cytb* | L43047 | Austin 1996 |  |
| *Puffinus* | *lherminieri* | *cytb* | U57815 | Austin 1996 |  |
| *Puffinus* | *pacificus* | *cox1* | JF498895.1 | Kerr et al. 2001 | USNM:643456 |
| *Puffinus* | *pacificus* | *cytb* | AF076088.1 | Nunn & Stanley 1998 |  |

b.

Supplementary Material 2: Primer sequences, PCR profile and conditions

b.

Shearwater-specific primers were designed using Primer3 (Untergasser et al. 2012) to amplify and sequence the three mitochondrial markers and *irf2*, *rag1* and *tpm*. We used primers designed from avian genomes to amplify *pax* and *csde* (Kimball et al. 2009). Due to their short size and low level of polymorphism, *pax*, *tpm* and *irf2* were sequenced only in one direction (forward strand). Because of issues of length polymorphism resulting in unreadable chromatograms, we designed internal primers for the *βfib* marker, in addition to the two designed by Gangloff et al. (2013). Polymerase Chain Reactions (PCR) were carried out using TaKaRa ExTaq® Polymerase Hot-Start Version, in a total volume of 30 µL, using 1X Ex Taq Buffer (Mg2+ plus) with a final concentration of 200 µM of dNTP, 0.8 µM of each primer, 0.015 U of Taq and 60 ng of DNA extract. For all markers, after an initial denaturation step of two min at 95°C, we ran 40 PCR cycles consisting in 1 min at 95°C, 1 min at the primer-specific annealing temperature (varying between 52°C and 64°C) and 1 min at 72°C. These cycles were followed by a 7 min final extension step at 72°C. PCR products were purified and sequenced by Eurofins Genomics Munich. Chromatograms were checked and assembled into contigs using Sequencher v.5.4.1 (Gene Codes Corporation).

| Primer name | Sequence | Origin | Strand | Marker | Melting temperature |
| --- | --- | --- | --- | --- | --- |
| Co1-F1-PUF-CRI | CTCAGCCTACTCATCCGTG | this study | Forward | *cox1* | 58.8°C |
| Co1-R3-PUF-CRI | TGTTGRTATAGGACTGGGTC | this study | Reverse | *cox1* | 56.3°C |
| Cytb-F1-Puf-CRI | GGCCTACTACTAGCYATACA | this study | Forward | *cytb* | 56.3°C |
| Cytb-R4-PUF-CRI | GTTARGATGAATAGGTTRGCG | this study | Reverse | *cytb* | 55.9°C |
| RCM-PUF-CRI-F | GGGTTGCTGATTTCTCGTGA | this study | Forward | Control region | 57.3°C |
| RCM-PUF-CRI-R | GGCAAACACATTCAATGCATG | this study | Reverse | Control region | 55.9°C |
| PAX-20F | CCCTCAGACACTGGATTAYGAATCAT | Kimball et al. 2009 | Forward | *pax* | 62.4°C |
| PAX-21R | CCAAGGATTCCGAAGCAGTAAG | Kimball et al. 2009 | Reverse | *pax* | 60.3°C |
| CSDE-5F | CTGGTGCTGTAAGTGCTCGTAAC | Kimball et al. 2009 | Forward | *csde* | 64.6°C |
| CSDE-6R | CCAGGCTGTAAGGTTTCTAGGTCAC | Kimball et al. 2009 | Reverse | *csde* | 62.4°C |
| TPM-F1-CRI | TGCAACCCAAGTCTTTCAGC | this study | Forward | *tpm* | 57.3°C |
| TPM-R2-CRI | TTCGGAAGGAAGGCAGGAAA | this study | Reverse | *tpm* | 57.3°C |
| IRF2-F-PUF-CRI | TGAAATTGAAAACCTAAGGCGAA | this study | Forward | *irf2* | 55.3°C |
| IRF2-R-PUF-CRI | TGGAACTCTCTTTCTGCAGGA | this study | Reverse | *irf2* | 55.9°C |
| BFIB-BI7U | GGAGAAAACAGGACAATGACAATTCAC | Gangloff et al. 2013 | Forward | *βfib* (first half) | 61.9°C |
| BFIB-R2-CRI | ACAATTGAGCTCCTGTCTTCTG | this study | Reverse | *βfib* (first half) | 58.4°C |
| BFIB-BI7L | TCCCCAGTAGTATCTGCCATTAGGGTT | Gangloff et al. 2013 | Forward | *βfib* (second half) | 64.6°C |
| BFIB-F3-CRI | CAGAAGACAGGAGCTCAATTGT | this study | Reverse | *βfib* (second half) | 58.4°C |
| RAG1-PUF-CRI-F | TCGCTCCAGATTTTCAGCATG | this study | Forward | *rag1* | 57.3°C |
| RAG1-PUF-CRI-R | TCTTGCCAGATCTGTGAGCA | this study | Reverse | *rag1* | 57.9°C |

Supplementary material 3: Detection and removal of nuclear pseudogenes of mitochondrial origin (numts)

To avoid numts, we digested nuclear DNA with the ExonucleaseV (ExoV; NEB-M0345S) prior to PCR amplification and Sanger sequencing of mitochondrial markers, using the following protocol inspired by (Jayaprakash et al. 2015). One ng of DNA sample was heated to 70°C to inactivate any residual Proteinase K from the extraction protocol. Digestion was then carried out, adding to the sample 1X NEB4 Buffer, 1 mM ATP, 0.3 U of ExoV, and 0.24 mg/mL of BSA. The mix was heated to 37°C during 48h, followed by 30 min at 70°C to inactivate the exonuclease.

To test whether the digestion protocol effectively removed all traces of nuclear DNA, we performed PCR amplifications on the same three individuals before and after ExoV digestion, targeting a nuclear marker (*pax*; 515 bp) and a mitochondrial marker (*cytb;* 877 bp). Our expectation was that ExoV treatment would destroy all template for *pax* amplification, while sparing mtDNA. We compensated the lowered PCR yield by using BSA at a final concentration of 0.24 mg/mL. PCR products were sent to Eurofins for sequencing. The results on an agarose gel are showed here (a.). The three individuals are I008058 (*baroli*), 142212 (*bailloni*) and BW51 (*nicolae*). D1, D2 and D3 correspond to the same individual after applying the digestion protocol. The two markers indicated here are *pax* and *cytb*. As expected, the nuclear marker is not amplified after digestion, whereas the mitochondrial marker is still amplified for the three individuals. Below are showed the chromatograms before (b.) and after the digestion step (c.) of *cox1* marker base 198 to 291 from 142208 (*bailloni*) individual. The double peaks present in the chromatograms before digestion are not present after the digestion step (position indicated by arrows).


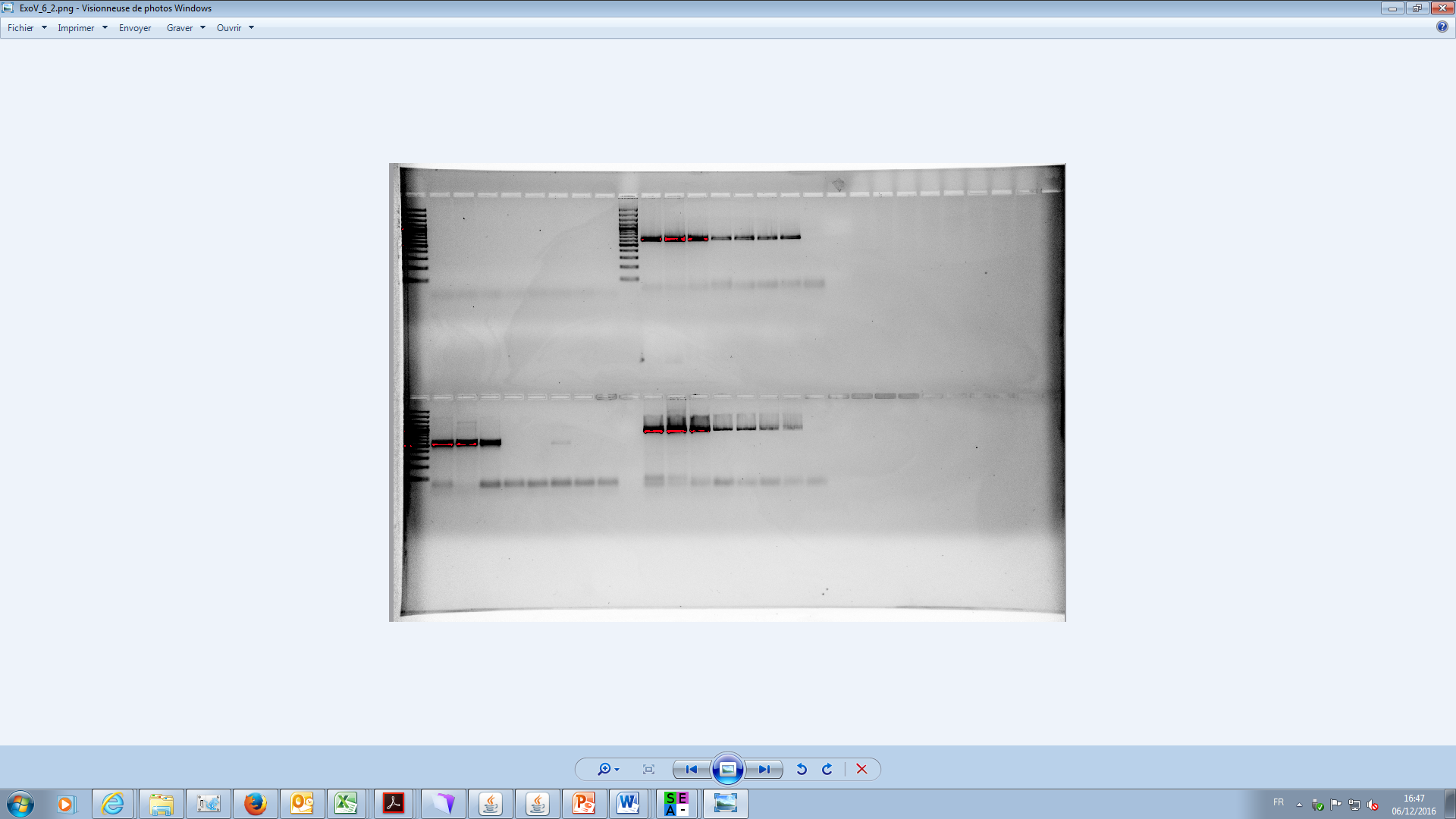

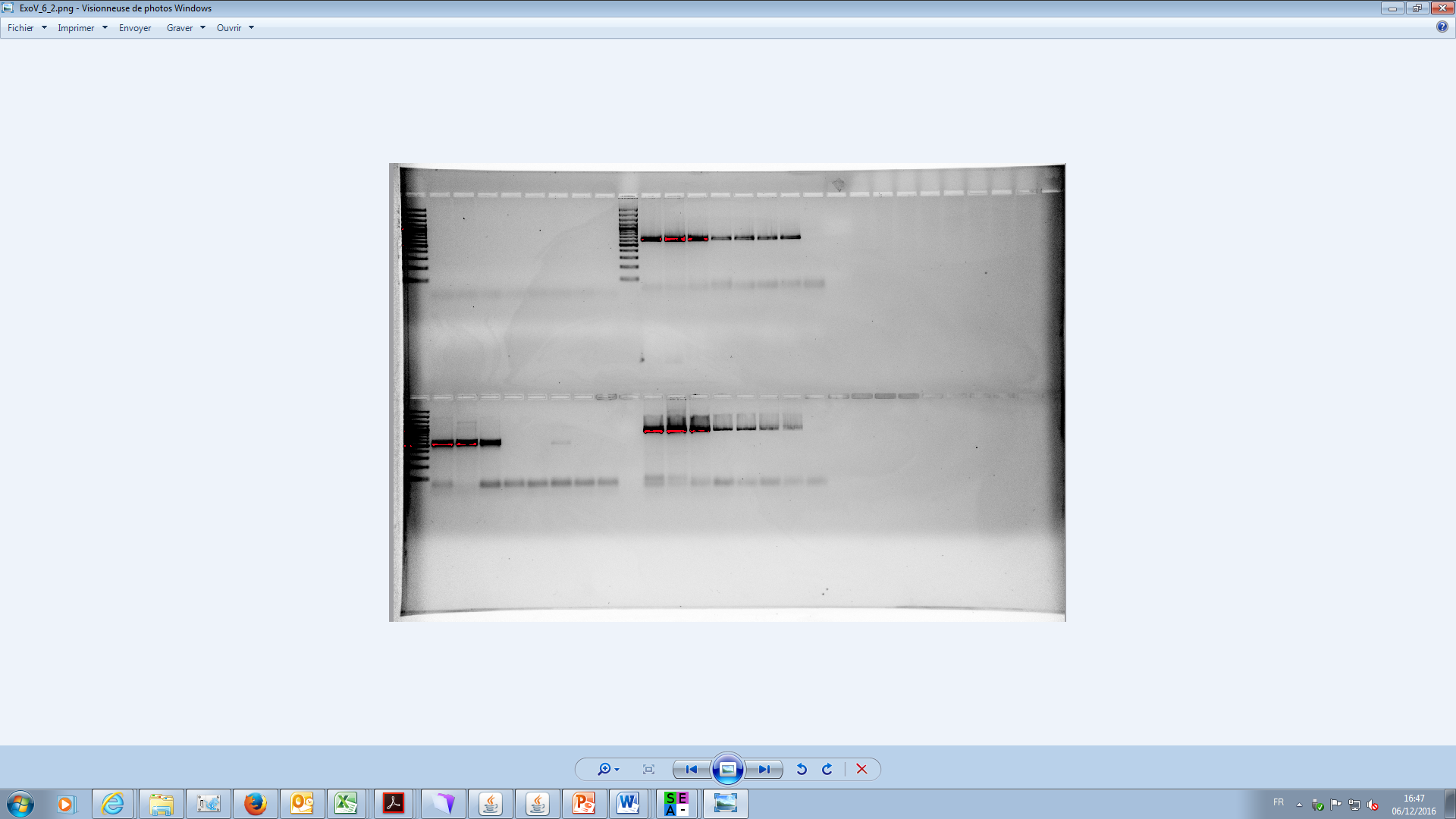

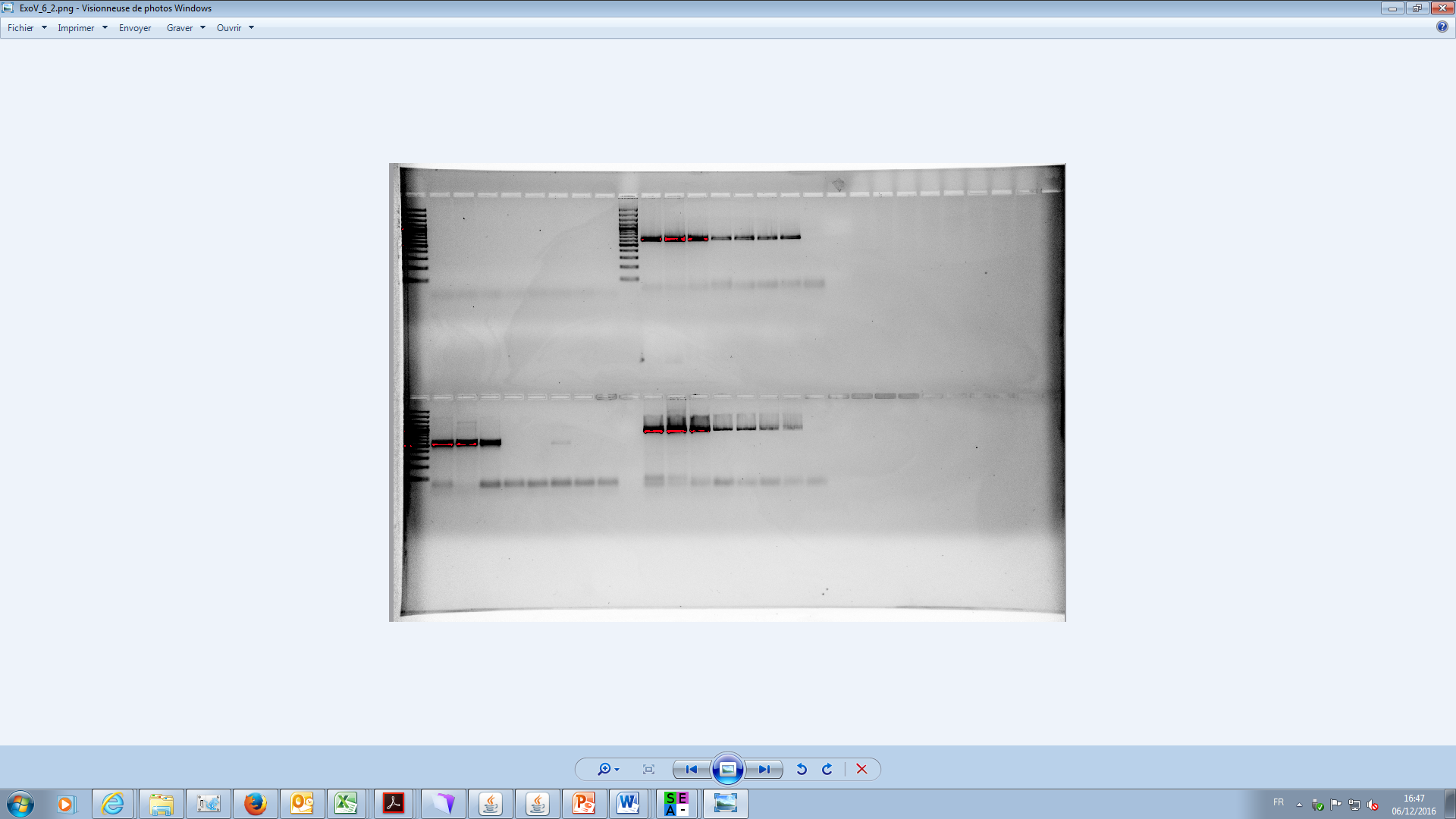


*pax*

*cytb*

I008058

142212

BW51

D1

D2

D3


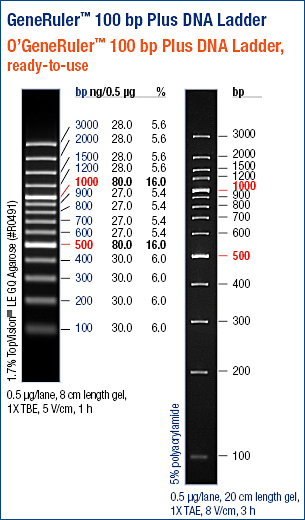


Blank

I008058

142212

BW51

D1

D2

D3

Blank

a.


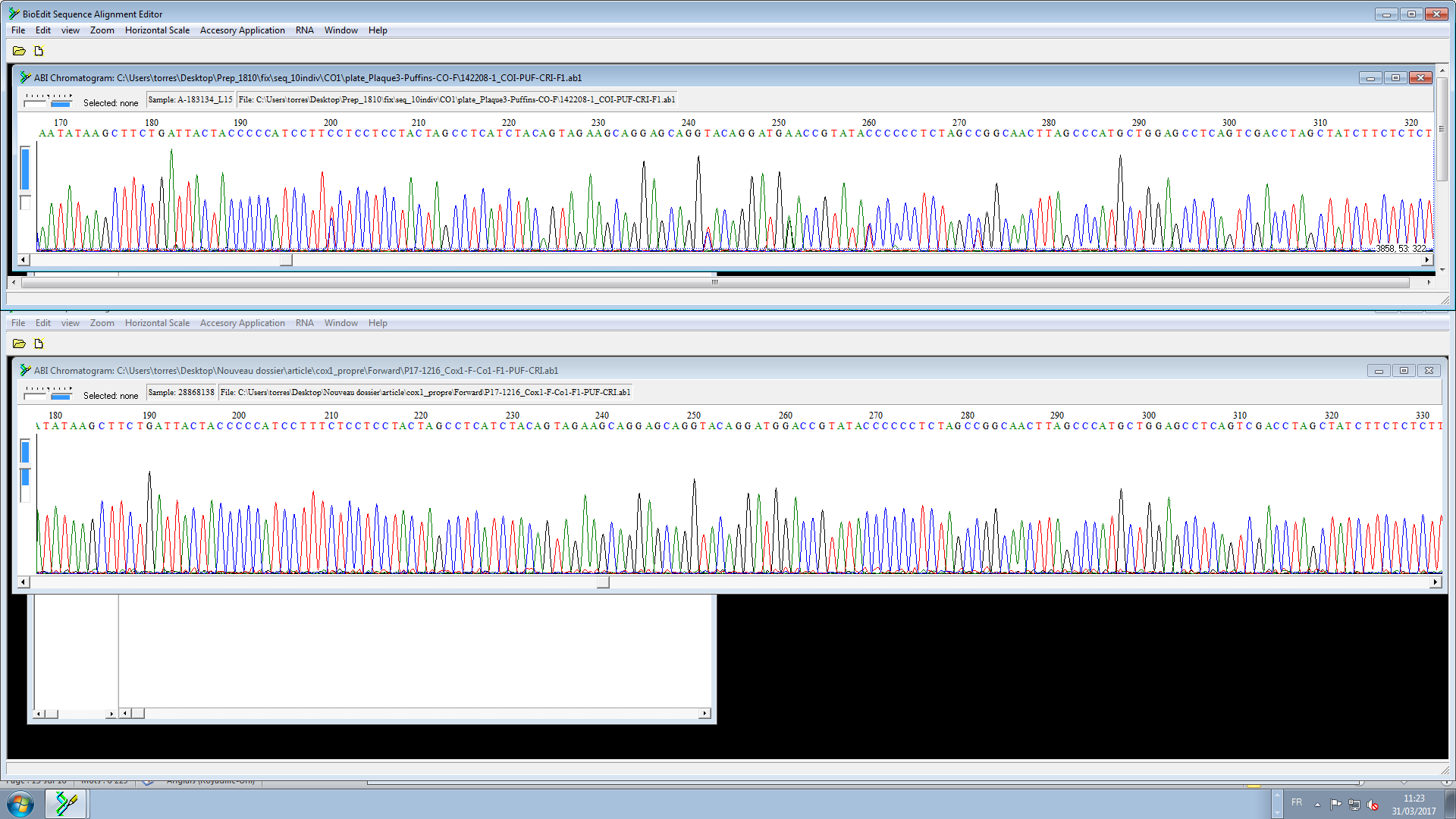

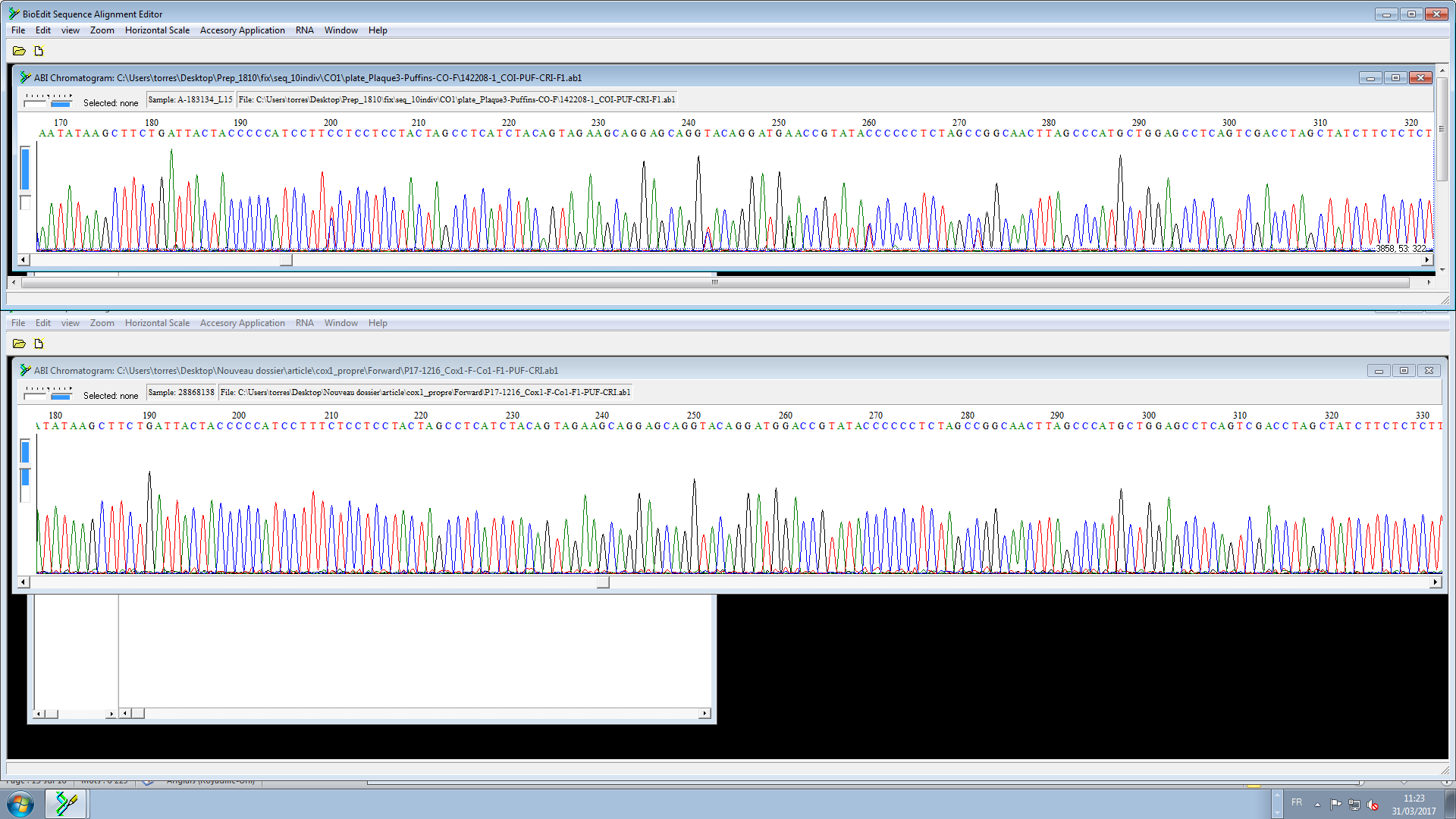


b.

Supplementary Material 4: Bayesian inference of gene trees and species tree

Among- and within lineage divergence, as well as reciprocal monophyly were evaluated using gene trees inferred using MrBayes v 3.2.6 (Ronquist et al. 2012a). We ran two independents MCMC chains of 50x10^6 generations each, sampled every 100 generations, using three heated and one cold chain. The first 25% were discarded as burn-in. We used the model resulting from jModelTest2 (Darriba , Taboada, Dollado, & Posada, 2015), with *Puffinus pacificus* as an outgroup for mitochondrial markers (Genbank sequences: JF498895.1 for *cox1* (Kerr et al. 2007) and AF076088.1 for *cytb* (Nunn & Stanley 1998) and sequences that we obtained of *Puffinus pacificus* for nuclear markers. We investigated the stationarity, visualizing the log-likelihood across generations in Tracer v 1.6 (Rambaut & Drummond 2007), checking the Effective Sample Size (ESS) and the convergence of multiple independent runs. Trees were inferred for all mitochondrial markers, and for both mitochondrial and nuclear markers, each marker considered independently. For each marker, the optimal model of substitution was estimated by jModeltest. These models were also inputted in both *BEAST and DiyABC. For each gamma model, four categories were inputted. For *BEAST, substitution rates were fixed to 1 for each marker. For DiyABC all population size priors followed a uniform distribution between 10^3^ and 10^5^ individuals, all times of divergence priors followed a uniform distribution between 10^4^ and 2.10^6^ years ago.

| Marker | Gamma shape | Proportion of invariant sites | Substitution model | Model param | Model param | Model param | Model param | Model param | Model param |
| --- | --- | --- | --- | --- | --- | --- | --- | --- | --- |
| *cox1* | 0.02 | 0.8 | HKY | 11.5 |  |  |  |  |  |
| *cytb* | 1 | 0.8 | TN93 | 3.2 | 5.8 |  |  |  |  |
| CR | 0.75 | 0.45 | GTR | 0.1 | 14.3 | 0.1 | 1 | 7.4 | 1 |
| *pax* | 1 | 0.5 | HKY | 1.88 |  |  |  |  |  |
| *csde* | 0.5 | 0.9 | TN93 | 4.6 | 4.7 |  |  |  |  |
| *tpm* | 0.5 | 0.5 | HKY | 8.7 |  |  |  |  |  |
| *irf2* | 0.5 | 0.5 | HKY | 4.2 |  |  |  |  |  |
| *βfib* | 0.5 | 0.8 | TN93 | 3.2 | 5.8 |  |  |  |  |
| *rag1* | 0.5 | 0.9 | GTR | 1 | 2 | 0.2 | 0.2 | 2 | 1 |

Supplementary material 5: Divergence scenario inferred by DiyABC

We used a coalescent-based ABC approach to explore the demographic scenario best describing the dataset of the combined mitochondrial and nuclear markers using the program DIYABC v. 2.1.0 (Cornuet et al. 2014). ABC methods consist in the simulation of datasets similar to the real dataset in terms of population and marker sizes. For each possible scenario, 10^6^ pseudo-observed datasets were simulated, with the same ploidy and number of loci per population as observed in the real dataset. We fixed uniform priors for population sizes and divergence times priors. All population size priors followed a uniform distribution between 10^3^ and 10^5^ individuals, all times of divergence priors followed a uniform distribution between 10^4^ and 2.10^6^ years ago. For each marker, the optimal model of substitution was estimated by jModeltest. These models were inputted in both *BEAST and DiyABC. For each gamma model, four categories were inputted. We simulated the datasets with these parameters. Summary statistics were calculated from the simulated datasets and compared to the same statistics obtained from the real dataset. The Euclidean distance was calculated between the statistics obtained for each normalized simulated dataset and those for the observed dataset (Beaumont et al. 2002). Posterior probability of each scenario was then calculated using a logistic regression on summary statistics produced by the 1% of the simulated datasets the closest to the real dataset based on this Euclidian distance. To reduce the dimensionality of the data, a linear discriminant analysis was preliminarily applied to the summary statistics (Estoup et al. 2012). The scenario with the highest posterior probability value with a 95% confidence interval (95% CI) non-overlapping with other scenarios was selected.


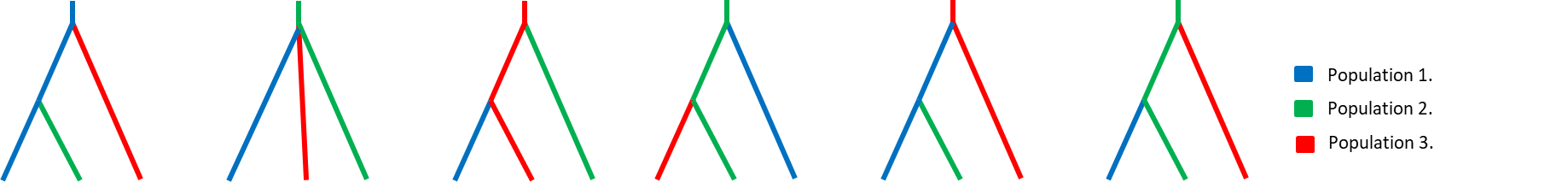
To test the evolutionary scenario including only two groups, only two scenarios were possible and tested. When the scenario included 3 or 4 populations, we ran a hierarchical analysis by comparing first the posterior probability given by different topologies including all the populations. The different topologies tested for 3 populations is presented as an example below (a.).

a.


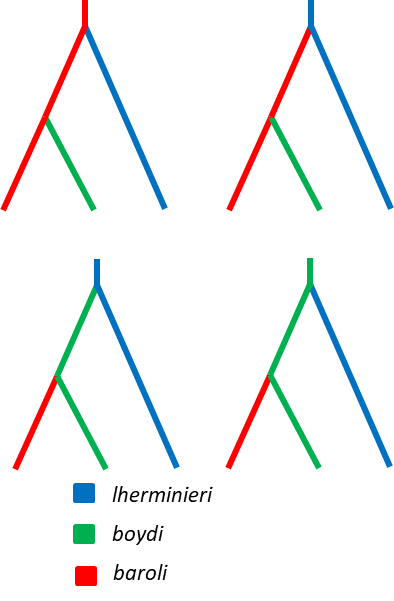
Once the best-fit topology was found, we compared the posterior probability given by different ancestral population for each node. The tested scenarios (with mitochondrial markers only) are represented for North Atlantic lineages, *lherminieri* populations, *baroli* populations and Indian Ocean populations in panel b.

b.

We present hereafter the best-fit scenario for all populations. In each case, the left scenario was inferred for all mitochondrial markers and the right scenario was inferred for all markers. Posterior probabilities of each scenario were calculated using a logistic regression on summary statistics produced by the 1% of the simulated datasets the closest to the real dataset. The 95% confidence intervals of probability of the selected scenario after 10000 samples of the regression are indicated. We selected scenarios which 95% confidence interval did not include 0. c. All populations. d. Atlantic lineages e. *lherminhieri* populations d. *baroli* populations g. Indian lineages. The selected scenarios show that Indian populations are ancestral over Atlantic populations. Mitochondrial markers show that *boydi* diverged from *lherminieri* whereas the use of both mitochondrial and nuclear markers show that *lherminieri* diverged from *boydi*. This incongruence is resolved later. Within *lherminieri* all markers show that Martinique (the southern) is the most ancestral population and Longcay (the northern) is the most recent. However we cannot determine if Longcay diverged from Allencay in a stepping stone process or from Martinique. Similarly within *baroli*, selvage population was the most ancestral but uncertainty remains in the order of appearance of the other populations. Within the Indian ocean, all markers show that *nicolae* is the ancestral lineage and the use of all marker show that the first population that have appeared is the North Reunion.

c.


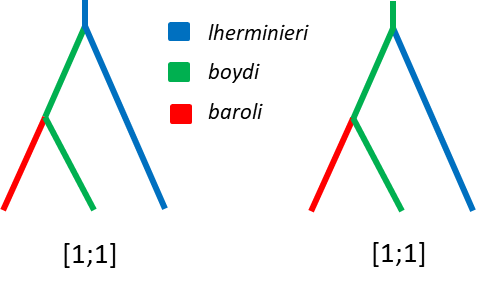

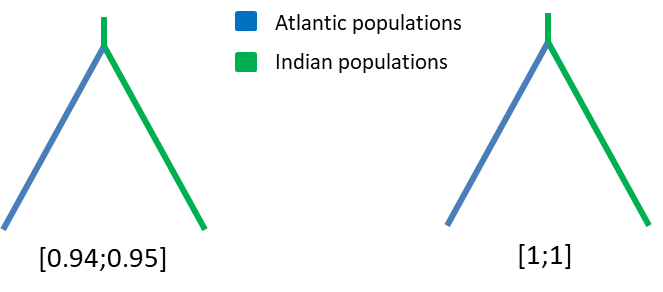


d.

f.

e.


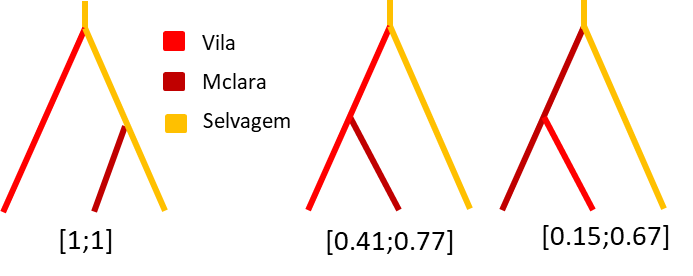

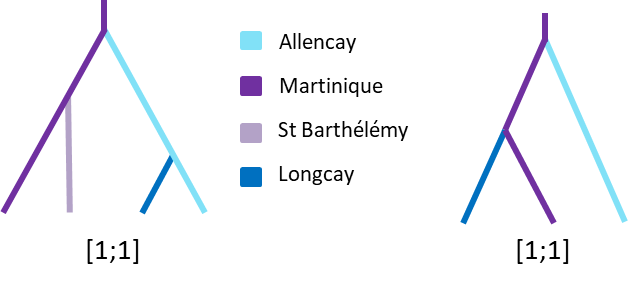


g.


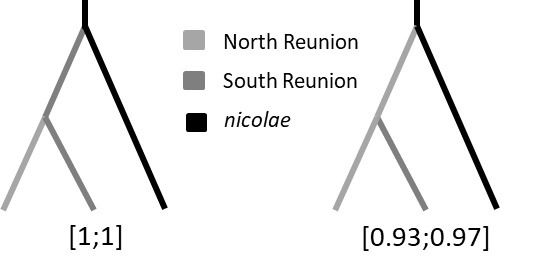


Finally we tried to define the population that originally colonized the Atlantic Ocean from the Indian Ocean (dashed branch in h.). We have previously defined that *nicolae* was the ancestral lineage in the Indian Ocean and that either *boydi* either *lherminieri* was the ancestral lineage in the Atlantic Ocean. We tested if the ancestral lineage in the Atlantic Ocean was either *boydi*, *lherminieri*,or *nicolae*. The hypothesis of *nicolae* being the ancestral lineage in the Atlantic ocean i.e. the dashed line being black could not be tested as such in the Diyabc software. We tested the hypothesis of the two Atlantic lineages as appearing simultaneously from *nicolae* as an equivalent. Results are presented in the lower panel of h. The selected scenario is considering *nicolae* as the ancestral lineage in the Atlantic Ocean.


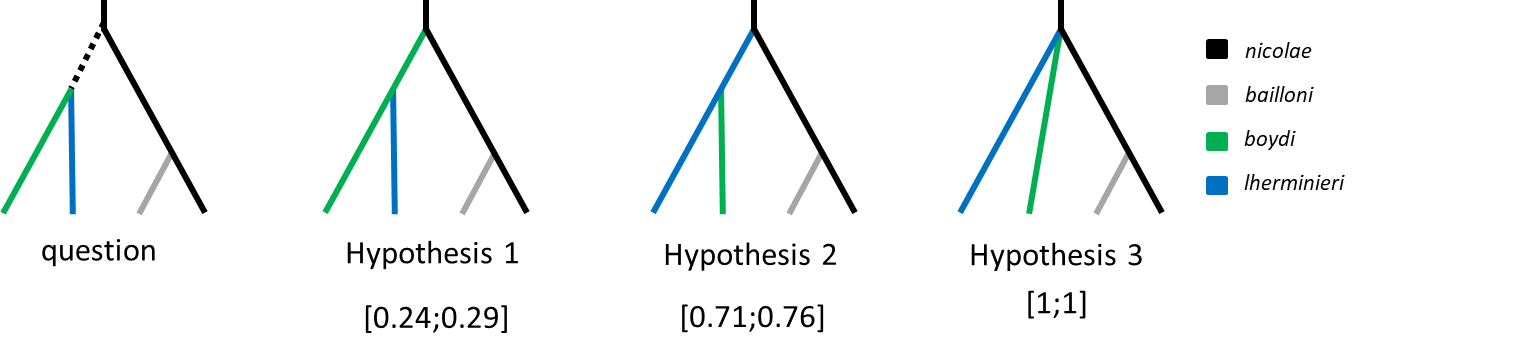


h.

Supplementary material 6: Comparative analysis of the impact of the supplemental CR sequences

Due to the presence of numts and a duplicated region, we observed the presence of double peaks in the mitochondrial CR sequences (see Supplementary Material 3 for the removal of numts). As these sequences represented a third of the CR dataset, we decided to evaluate the effect of noise in phylogeographic analyses. We made all the Φ_ST_, Tajima’s D, Fu’s Fs, MrBayes and *BEAST analyses using three datasets. The first dataset included all mitochondrial markers and all individuals. In this dataset, CR sequences either contained ambiguities (the Φ_ST_, Tajima’s D, Fu’Fs analyses) or were phased (MrBayes and *BEAST analyses). The second dataset contained only ambiguity-free *cox1* and *cytb* sequences. The third dataset was built by removing from the first dataset all 60 individuals that displayed double-peaks at the CR locus (*BEAST analyses), or for CR sequences only, encoding missing data for that locus (Arlequin and MrBayes analyses). *BEAST analyses were run for on mitochondrial markers only and on mitochondrial and nuclear markers. Dropping the CR marker altogether resulted in a strong loss of information. Most Φ_ST_ values are strongly higher in the analysis of the three mitochondrial markers than in the analysis on only two (Table S1).

| a. |  | ***lherminieri*** | ***lherminieri*** | ***lherminieri*** | ***lherminieri*** | ***boydi*** | ***boydi*** | ***baroli*** | ***baroli*** | ***baroli*** | ***baroli*** | ***bailloni*** | ***bailloni*** | ***nicolae*** |
| --- | --- | --- | --- | --- | --- | --- | --- | --- | --- | --- | --- | --- | --- | --- |
|  |  | **Allencay** | **Longcay** | **Martinique** | **St**  **Barthélémy** | **Raso** | **Cima** | **Funchal** | **Mclara** | **Selvagem** | **Vila** | **North**  **Reunion** | **South**  **Reunion** | **Seychelles** |
| ***lherminieri*** | **Allencay** | - |  |  |  |  |  |  |  |  |  |  |  |  |
| ***lherminieri*** | **Longcay** | 0.08* | - |  |  |  |  |  |  |  |  |  |  |  |
| ***lherminieri*** | **Martinique** | 0 | 0.22*** | - |  |  |  |  |  |  |  |  |  |  |
| ***lherminieri*** | **St**  **Barthélémy** | 0.19* | 0.35*** | 0 | - |  |  |  |  |  |  |  |  |  |
| ***boydi*** | **Raso** | 0.81*** | 0.83*** | 0.82*** | 0.77*** | - |  |  |  |  |  |  |  |  |
| ***boydi*** | **Cima** | 0.76*** | 0.78*** | 0.74*** | 0.71*** | 0 | - |  |  |  |  |  |  |  |
| ***baroli*** | **Funchal** | 0.81*** | 0.84*** | 0.85*** | 0.72*** | 0.7*** | 0.56*** | - |  |  |  |  |  |  |
| ***baroli*** | **Mclara** | 0.78*** | 0.81*** | 0.82*** | 0.73*** | 0.67*** | 0.61*** | 0 | - |  |  |  |  |  |
| ***baroli*** | **Selvagem** | 0.8*** | 0.83*** | 0.83*** | 0.73*** | 0.64*** | 0.53*** | 0 | 0 | - |  |  |  |  |
| ***baroli*** | **Vila** | 0.85*** | 0.86*** | 0.84*** | 0.81*** | 0.69*** | 0.65*** | 0 | 0 | 0 | - |  |  |  |
| ***bailloni*** | **North**  **Reunion** | 0.93*** | 0.93*** | 0.89*** | 0.91*** | 0.87*** | 0.84*** | 0.89*** | 0.85*** | 0.88*** | 0.89*** | - |  |  |
| ***bailloni*** | **South**  **Reunion** | 0.93*** | 0.94*** | 0.9*** | 0.92*** | 0.89*** | 0.87*** | 0.91*** | 0.88*** | 0.88*** | 0.91*** | 0 | - |  |
| ***nicolae*** | **Seychelles** | 0.93*** | 0.93*** | 0.9*** | 0.91*** | 0.89*** | 0.87*** | 0.91*** | 0.89*** | 0.9*** | 0.92*** | 0.76*** | 0.79*** | - |

| b. |  | ***lherminieri*** | ***lherminieri*** | ***lherminieri*** | ***lherminieri*** | ***boydi*** | ***boydi*** | ***baroli*** | ***baroli*** | ***baroli*** | ***baroli*** | ***bailloni*** | ***bailloni*** | ***nicolae*** |
| --- | --- | --- | --- | --- | --- | --- | --- | --- | --- | --- | --- | --- | --- | --- |
|  | . | **Allencay** | **Longcay** | **Martinique** | **St**  **Barthélémy** | **Raso** | **Cima** | **Funchal** | **Mclara** | **Selvagem** | **Vila** | **North**  **Reunion** | **South**  **Reunion** | **Seychelles** |
| ***lherminieri*** | **Allencay** | - |  |  |  |  |  |  |  |  |  |  |  |  |
| ***lherminieri*** | **Longcay** | 0 | - |  |  |  |  |  |  |  |  |  |  |  |
| ***lherminieri*** | **Martinique** | 0 | 0.26*** | - |  |  |  |  |  |  |  |  |  |  |
| ***lherminieri*** | **St**  **Barthélémy** | 0.21* | 0.45*** | 0 | - |  |  |  |  |  |  |  |  |  |
| ***boydi*** | **Raso** | 0.89*** | 0.91*** | 0.92*** | 0.92*** | - |  |  |  |  |  |  |  |  |
| ***boydi*** | **Cima** | 0.89*** | 0.91*** | 0.91*** | 0.92*** | 0 | - |  |  |  |  |  |  |  |
| ***baroli*** | **Funchal** | 0.88*** | 0.91*** | 0.94*** | 0.91*** | 0.86*** | 0.85*** | - |  |  |  |  |  |  |
| ***baroli*** | **Mclara** | 0.9*** | 0.91*** | 0.92*** | 0.92*** | 0.85*** | 0.85*** | 0 | - |  |  |  |  |  |
| ***baroli*** | **Selvagem** | 0.88*** | 0.9*** | 0.94*** | 0.92*** | 0.87*** | 0.86*** | 0 | 0 | - |  |  |  |  |
| ***baroli*** | **Vila** | 0.86*** | 0.9** | 0.94** | 0.91** | 0.85*** | 0.84*** | 0 | 0 | 0 | - |  |  |  |
| ***bailloni*** | **North**  **Reunion** | 0.94*** | 0.95*** | 0.93*** | 0.96*** | 0.96*** | 0.95*** | 0.95*** | 0.95*** | 0.95*** | 0.95*** | - |  |  |
| ***bailloni*** | **South**  **Reunion** | 0.95*** | 0.96*** | 0.94*** | 0.97*** | 0.97*** | 0.96*** | 0.96*** | 0.96*** | 0.96*** | 0.96*** | 0.05* | - |  |
| ***nicolae*** | **Seychelles** | 0.79*** | 0.8*** | 0.72*** | 0.78*** | 0.8*** | 0.81*** | 0.8*** | 0.83*** | 0.78*** | 0.75** | 0.54*** | 0.6*** | - |

Table S1. Pairwise Φ_ST_ values for a. all mitochondrial markers and all individuals, b. cox1 and *cytb* only for all individuals and c. all mitochondrial markers but individuals presenting ambiguities for the control region were removed. Border indicates the separation between intra and inter lineage. Triple band indicates the separation between intra- and inter-ocean comparisons. *: p<0.05; ***: p<0.001

| c. |  | ***lherminieri*** | ***lherminieri*** | ***lherminieri*** | ***lherminieri*** | ***boydi*** | ***boydi*** | ***baroli*** | ***baroli*** | ***baroli*** | ***baroli*** | ***bailloni*** | ***bailloni*** | ***nicolae*** |
| --- | --- | --- | --- | --- | --- | --- | --- | --- | --- | --- | --- | --- | --- | --- |
|  |  | **Allencay** | **Longcay** | **Martinique** | **St**  **Barthélémy** | **Raso** | **Cima** | **Funchal** | **Mclara** | **Selvagem** | **Vila** | **North**  **Reunion** | **South**  **Reunion** | **Seychelles** |
| ***lherminieri*** | **Allencay** | - |  |  |  |  |  |  |  |  |  |  |  |  |
| ***lherminieri*** | **Longcay** | 0 | - |  |  |  |  |  |  |  |  |  |  |  |
| ***lherminieri*** | **Martinique** | 0 | 0.3*** | - |  |  |  |  |  |  |  |  |  |  |
| ***lherminieri*** | **St**  **Barthélémy** | 0 | 0.48*** | 0 | - |  |  |  |  |  |  |  |  |  |
| ***boydi*** | **Raso** | 0.85*** | 0.86*** | 0.86*** | 0.85*** | - |  |  |  |  |  |  |  |  |
| ***boydi*** | **Cima** | 0.83*** | 0.84*** | 0.81*** | 0.82*** | 0 | - |  |  |  |  |  |  |  |
| ***baroli*** | **Funchal** | 0.83*** | 0.85*** | 0.86*** | 0.82*** | 0.71*** | 0.69*** | - |  |  |  |  |  |  |
| ***baroli*** | **Mclara** | 0.88*** | 0.89*** | 0.89*** | 0.89*** | 0.74*** | 0.73*** | 0 | - |  |  |  |  |  |
| ***baroli*** | **Selvagem** | 0.87*** | 0.89*** | 0.92*** | 0.91*** | 0.74*** | 0.67*** | 0 | 0 | - |  |  |  |  |
| ***baroli*** | **Vila** | 0.82*** | 0.83*** | 0.85*** | 0.78*** | 0.73*** | 0.64*** | 0 | 0 | 0 | - |  |  |  |
| ***bailloni*** | **North**  **Reunion** | 0.94*** | 0.95*** | 0.94*** | 0.96*** | 0.92*** | 0.9*** | 0.9*** | 0.94*** | 0.96*** | 0.89*** | - |  |  |
| ***bailloni*** | **South**  **Reunion** | 0.94*** | 0.94*** | 0.92*** | 0.95*** | 0.92*** | 0.91*** | 0.91*** | 0.94*** | 0.94*** | 0.92*** | 0 | - |  |
| ***nicolae*** | **Seychelles** | 0.92*** | 0.93*** | 0.91*** | 0.93*** | 0.91*** | 0.9*** | 0.91*** | 0.93*** | 0.93*** | 0.9*** | 0.78*** | 0.8*** | - |

Similarly, if no Fu’s Fs value was significant, regardless of the dataset, Cima, Mclara and North Reunion Tajima’s D values were significant using only two mitochondrial markers rather than three (Table S2).

Table S2. Results of Fu’s Fs and Tajima’s D for a. all mitochondrial markers and all individuals, b. *cox1* and *cytb* only for all individuals and c. all mitochondrial markers but individuals presenting ambiguities for the control region were removed.

| a. | Allencay | Longcay | Martinique | St  Barthélémy | Raso | Cima | Mclara | Vila | Selvagem | Funchal | North  Reunion | South  Reunion | Seychelles | Mean | s.d. |
| --- | --- | --- | --- | --- | --- | --- | --- | --- | --- | --- | --- | --- | --- | --- | --- |
| Tajima's D test |  |  |  |  |  |  |  |  |  |  |  |  |  |  |  |
| Sample size | 19 | 20 | 44 | 8 | 18 | 18 | 15 | 19 | 10 | 4 | 28 | 32 | 41 | 21 | 12 |
| S | 17 | 14 | 1 | 48 | 61 | 64 | 49 | 49 | 37 | 24 | 14 | 47 | 14 | 34 | 21 |
| Pi | 2053 | 1930 | 2302 | 1901 | 2279 | 1349 | 1914 | 1963 | 2845 | 2051 | 2495 | 2413 | 2543 | 2157 | 379 |
| Tajima's D | -0.47 | -0.27 | 7.39 | -2.59 | -2.23 | -0.61 | -0.95 | -1.35 | -2.40 | -1.12 | -1.18 | -2.40 | 0.34 | -0.60 | 2.57 |
| p-value | 0.352 | 0.419 | 1 | 0 | 0 | 0.285 | 0.193 | 0.071 | 0 | 0 | 0.104 | 0.002 | 0.718 | 0.24185 | 0.31477 |
| Fu's FS test |  |  |  |  |  |  |  |  |  |  |  |  |  |  |  |
| No. of alleles | 19 | 20 | 44 | 8 | 18 | 18 | 15 | 19 | 10 | 4 | 28 | 32 | 41 | 21 | 12 |
| Theta_pi | 2053 | 1930 | 2302 | 1901 | 2279 | 1349 | 1914 | 1963 | 2845 | 2051 | 2495 | 2413 | 2543 | 2157 | 379 |
| Exp. no. of alleles | 19 | 20 | 44 | 8 | 18 | 18 | 15 | 19 | 10 | 4 | 28 | 32 | 41 | 21 | 12 |
| FS | 2.45 | 2.27 | 0.68 | 4.21 | 2.67 | 2.12 | 2.88 | 2.40 | 4.14 | 5.83 | 1.81 | 1.48 | 0.97 | 2.61 | 1.42 |
| p-value | 0.624 | 0.602 | 0.537 | 0.731 | 0.722 | 0.533 | 0.599 | 0.624 | 0.834 | 0.608 | 0.752 | 0.667 | 0.658 | 0.65315 | 0.08694 |

| b. | Allencay | Longcay | Martinique | St  Barthélémy | Raso | Cima | Mclara | Vila | Selvagem | Funchal | North  Reunion | South  Reunion | Seychelles | Mean | s.d. |
| --- | --- | --- | --- | --- | --- | --- | --- | --- | --- | --- | --- | --- | --- | --- | --- |
| Tajima's D test |  |  |  |  |  |  |  |  |  |  |  |  |  |  |  |
| Sample size | 17 | 19 | 25 | 8 | 16 | 18 | 14 | 19 | 8 | 3 | 25 | 30 | 36 | 18.31 | 9.19 |
| S | 13 | 10 | 3 | 6 | 9 | 12 | 4 | 5 | 6 | 5 | 9 | 12 | 7 | 7.77 | 3.3 |
| Pi | 292.24 | 348.43 | 551.72 | 269.89 | 513.18 | 409.56 | 437.45 | 384.96 | 687.46 | 931.67 | 443.28 | 321.19 | 481.76 | 467.14 | 180.33 |
| Tajima's D | -1.04 | -0.84 | 0.1 | -0.85 | -2.09 | -2 | 0.79 | 1.24 | -2.14 | 0 | -1.77 | -2.23 | 11.18 | 0.03 | 3.55 |
| p-value | 0.16 | 0.21 | 0.63 | 0.23 | 0.01 | 0.01 | 0.79 | 0.89 | 0 | 1 | 0.02 | 0 | 1 | 0.38 | 0.41 |
| Fu's FS test |  |  |  |  |  |  |  |  |  |  |  |  |  |  |  |
| No. of alleles | 17 | 19 | 25 | 8 | 16 | 18 | 14 | 19 | 8 | 3 | 25 | 30 | 36 | 18.31 | 9.19 |
| Theta_pi | 292.24 | 348.43 | 551.72 | 269.89 | 513.18 | 409.56 | 437.45 | 384.96 | 687.46 | 931.67 | 443.28 | 321.19 | 481.76 | 467.14 | 180.33 |
| Exp. no. of alleles | 16.55 | 18.53 | 24.47 | 7.9 | 15.77 | 17.64 | 13.8 | 18.57 | 7.96 | 3 | 24.35 | 28.72 | 34.75 | 17.85 | 8.81 |
| FS | 9.93 | 4.5 | 0 | 2.22 | 9.76 | 7.93 | 6.99 | 2.63 | 7.32 | 5.74 | 10.41 | 4.87 | 20.8 | 0.8 | 0.4 |
| p-value | 1 | 0.97 | 1 | 0.52 | 1 | 1 | 1 | 0.83 | 1 | 0.84 | 1 | 0.98 | 1 | 0.93 | 0.14 |

| c. | Allencay | Longcay | Martinique | St  Barthélémy | Raso | Cima | Mclara | Vila | Selvagem | Funchal | North  Reunion | South  Reunion | Seychelles | Mean | s.d. |
| --- | --- | --- | --- | --- | --- | --- | --- | --- | --- | --- | --- | --- | --- | --- | --- |
| Tajima's D test |  |  |  |  |  |  |  |  |  |  |  |  |  |  |  |
| Sample size | 19 | 20 | 26 | 8 | 18 | 18 | 15 | 19 | 8 | 4 | 18 | 33 | 36 | 18.62 | 9.22 |
| S | 13 | 10 | 3 | 6 | 35 | 44 | 28 | 5 | 6 | 22 | 15 | 15 | 7 | 16.08 | 12.72 |
| Pi | 618 | 579 | 681 | 416 | 769 | 546 | 639 | 534 | 857 | 926 | 388 | 606 | 620 | 629 | 154 |
| Tajima's D | -0.9 | -0.51 | 1.17 | -0.42 | -2.64 | -2.01 | -1.54 | 5.43 | -0.85 | -1.28 | -2.91 | -1.87 | 1.14 | -0.55 | 2.18 |
| p-value | 0.2 | 0.34 | 0.87 | 0.37 | 0 | 0.01 | 0.04 | 1 | 0.24 | 0 | 0 | 0.01 | 0.87 | 0.3 | 0.37 |
| Fu's FS test |  |  |  |  |  |  |  |  |  |  |  |  |  |  |  |
| No. of alleles | 19 | 20 | 26 | 8 | 18 | 18 | 15 | 19 | 8 | 4 | 18 | 33 | 36 | 18.62 | 9.22 |
| Theta_pi | 618 | 579 | 681 | 416 | 769 | 546 | 639 | 534 | 857 | 926 | 388 | 606 | 620 | 629 | 154 |
| Exp. no. of alleles | 18.73 | 19.68 | 25.53 | 7.93 | 17.8 | 17.73 | 14.84 | 18.69 | 7.97 | 3.99 | 17.62 | 32.16 | 35.02 | 18.28 | 8.94 |
| FS | 15.76 | 3.18 | 25.69 | 2.67 | 10.3 | 1.14 | 1.73 | 0.99 | 7.76 | 5.04 | 7.73 | 1.36 | 4.15 | 6.73 | 7.14 |
| p-value | 1 | 0.9 | 1 | 0.57 | 1 | 0.48 | 0.63 | 0.5 | 1 | 0.73 | 1 | 0.72 | 0.98 | 0.81 | 0.21 |

If the topology of the Bayesian tree is the same in the three analyses, posterior probabilities values at lineage nodes are lower using only two markers (Dataset 2; Fig. S1). The analyses based on datasets 3 led to results closer to the dataset 1. Φ_ST_ values are quite similar between the two datasets except for some intra-lineage values that are significant in one case and not in the other (Table S1). Again there were differences in the Tajima’s D values but not in the Fu’s Fs values (Table S2). The differences observed between the analyses of the datasets 1 and 3 mainly include the populations with fewer individuals (St-Barthélémy, Selvagem and Funchal). This could be due to a loss of statistical power when too many individuals are removed. Posterior probabilities values are highly similar between the two bayesian trees.

Figure S1. MrBayes trees for a. all mitochondrial markers and all individuals, b. *cox1* and *cytb* only for all individuals and c. all mitochondrial markers but individuals presenting ambiguities for the control region were removed. Posterior probabilities superior to 0.75 are showed. The scale bars show how the length of a branch translates in sequence divergence. The unit is divergent nucleotides divided by the length of the sequence analysed.

b.


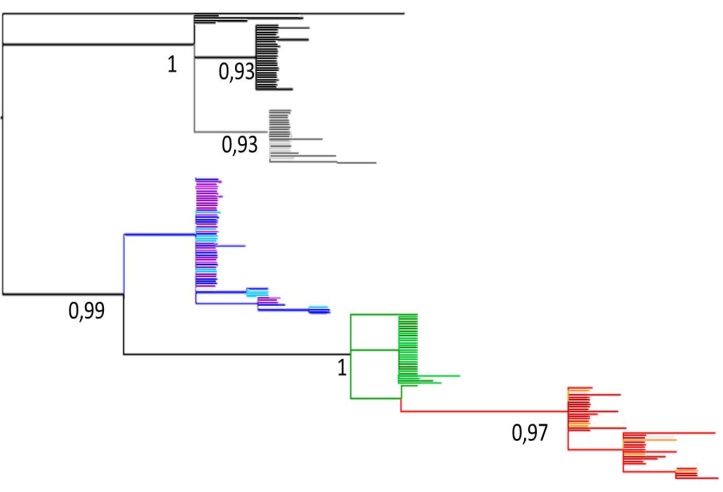


a.


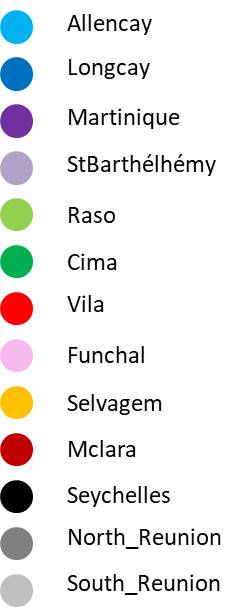


c.

However the divergence times between the lineages in the *BEAST analysis seems overestimated using dataset 1 (Table S3). This could be due to the supplemental information brought by the multiple copies of the control region in the dataset. This difference among the divergence time is lowered using also all the nuclear markers and the information they bring.

The artificial diversity brought by the duplicated sequences seems not bring major bias in the analyses, since the difference in the results are low when these sequences are removed and could be due to a loss of statistical power. These differences are lowered when adding supplemental markers to the analyses. We consider that the analyses performed using dataset 1 are valid.

|  | Atlantic/Indian split | East Atlantic/West Atlantic split | *boydi*/*baroli* split | *nicolae*/*bailloni* split |
| --- | --- | --- | --- | --- |
| Three mt markers, all individuals | 1.76 [0.99-2.60] | 1.38 [0.78-2.04] | 0.85 [0.44-1.32] | 0.72 [0.33-1.13] |
| Three mt markers, all individuals except 60 | 2.31 [1.38-3.32] | 1.72 [1.05-2.45] | 0.98 [0.55-1.43] | 0.62 [0.32-0.96] |
| *cox1* and *cytb* only | 1.96 [1.11-3.02] | 1.51 [0.84-2.13] | 0.90 [0.51-1.35] | 0.65 [0.33-1.10] |
| all markers, all individuals | 2.71 [1.17-4.72] | 1.90 [0.85-3.23] | 1.26 [0.54-2.18] | 1.01 [0.42-1.75] |
| all markers, all individuals except 60 | 2.80 [1.24-4.90] | 2.01 [1.05-3.44] | 1.25 [0.55-2.23] | 1.06 [0.52-1.83] |
| all markers except CR | 3.51 [2.54-4.45] | 1.49 [1.35-2.73] | 0.88 [0.51-1.25] | 0.80 [0.46-1.18] |

Table S3. *BEAST estimations of divergence times, for the three datasets defined in Suppl. M6. Median divergence time and their 95% confidence interval are showed.

Supplementary Material 7: Pairwise distance results and Fu’Fs and Tajima’s D results

Population average pairwise differences with above diagonal: average number of pairwise differences between populations (P_XY_), diagonal cells: average number of pairwise differences within population (P_X_), below diagonal: corrected average pairwise difference (P_XY_-(P_X_+P_Y_)/2)

a. All mitochondrial markers and all individuals. b. All nuclear markers and all individuals.

c. Results of Fu’s Fs and Tajima’s D for all mitochondrial markers and all individuals.

d. Results of Fu’s Fs and Tajima’s D for all nuclear markers and all individuals

Border indicates the separation between intra and inter lineage comparisons. Triple bands indicate the separation between intra- and inter-ocean comparisons.

*: p<0.05; ***:p<0.001

| a. |  | ***lherminieri*** | ***lherminieri*** | ***lherminieri*** | ***lherminieri*** | ***boydi*** | ***boydi*** | ***baroli*** | ***baroli*** | ***baroli*** | ***baroli*** | ***bailloni*** | ***bailloni*** | ***nicolae*** |
| --- | --- | --- | --- | --- | --- | --- | --- | --- | --- | --- | --- | --- | --- | --- |
|  |  | Allencay | Longcay | Martinique | St Barthélémy | Raso | Cima | Vila | Mclara | Selvagem | Funchal | North Reunion | South Reunion | Seychelles |
| ***lherminieri*** | Allencay | 4 | 4 | 4 | 8*** | 26*** | 27*** | 41*** | 35*** | 29*** | 31*** | 39*** | 48*** | 38*** |
| ***lherminieri*** | Longcay | 0 | 3 | 1*** | 2*** | 26*** | 27*** | 34*** | 28*** | 23*** | 22*** | 37*** | 45*** | 37*** |
| ***lherminieri*** | Martinique | 0 | 4*** | 3 | 6 | 23*** | 20*** | 33*** | 36*** | 29*** | 32*** | 22*** | 30*** | 26*** |
| ***lherminieri*** | St  Barthélémy | 1* | 9*** | -1 | 9 | 28*** | 28*** | 45*** | 29*** | 24*** | 26*** | 37*** | 49*** | 38*** |
| ***boydi*** | Raso | 32*** | 32*** | 29*** | 37*** | 8 | 10 | 26*** | 29*** | 23*** | 31*** | 30*** | 40*** | 36*** |
| ***boydi*** | Cima | 35*** | 34*** | 28*** | 39*** | 0 | 13 | 29*** | 30*** | 23*** | 29*** | 33*** | 43*** | 39*** |
| ***baroli*** | Vila | 35*** | 39*** | 27*** | 36*** | 18*** | 19*** | 8 | 0 | 0 | 0* | 38*** | 48*** | 47*** |
| ***baroli*** | Mclara | 27*** | 35*** | 28*** | 40*** | 19*** | 18*** | 9 | 11 | 11 | 13 | 31*** | 41*** | 41*** |
| ***baroli*** | Selvagem | 23*** | 29*** | 23*** | 33*** | 15*** | 13*** | 8 | 1 | 9 | 16 | 27*** | 32*** | 32*** |
| ***baroli*** | Funchal | 23*** | 30*** | 24*** | 37*** | 20*** | 16*** | 10 | 1 | 5 | 13 | 25*** | 39*** | 35*** |
| ***bailloni*** | North  Reunion | 42*** | 40*** | 25*** | 43*** | 35*** | 40*** | 43*** | 38*** | 32*** | 33*** | 2 | 2 | 10*** |
| ***bailloni*** | South  Reunion | 51*** | 48*** | 34*** | 55*** | 45*** | 51*** | 54*** | 48*** | 38*** | 47*** | 0 | 3 | 13*** |
| ***nicolae*** | Seychelles | 41*** | 40*** | 29*** | 43*** | 41*** | 47*** | 52*** | 48*** | 38*** | 42*** | 8*** | 11*** | 3 |

| b. |  | ***lherminieri*** | ***lherminieri*** | ***lherminieri*** | ***boydi*** | ***boydi*** | ***baroli*** | ***baroli*** | ***bailloni*** | ***bailloni*** | ***nicolae*** |
| --- | --- | --- | --- | --- | --- | --- | --- | --- | --- | --- | --- |
|  |  | Allencay | Longcay | Martinique | Raso | Cima | Vila | Mclara | North  Reunion | South  Reunion | Seychelles |
| ***lherminieri*** | Allencay | 0 | 0 | 2.72*** | 0 | 4.65*** | 3.93*** | 3.66*** | 5.34*** | 5.17*** | 4.32*** |
| ***lherminieri*** | Longcay | 0.05*** | 1.43*** | 1.95*** | 2.77*** | 3.53*** | 2.95*** | 3.4*** | 3.88*** | 4.58*** | 3.11*** |
| ***lherminieri*** | Martinique | 0 | 0 | 1.03*** | 0 | 2.47*** | 2.07*** | 3.65*** | 3.35*** | 3.93*** | 2.7*** |
| ***boydi*** | Raso | 0.39*** | 0.63*** | 0 | 0 | 0 | 0 | 0 | 0 | 0 | 0 |
| ***boydi*** | Cima | 1.28*** | 0.47*** | -0.39*** | 0 | 4.69*** | 0 | 0 | 0 | 0 | 0 |
| ***baroli*** | Vila | 1.78*** | 1.12*** | 0.44*** | 0 | 0 | 0 | 0 | 3.61*** | 4.76*** | 3.18*** |
| ***baroli*** | Mclara | 0*** | 1.06*** | 1.51*** | 0 | 0 | 0.23*** | 0 | 0 | 0 | 0 |
| ***bailloni*** | North  Reunion | 2.8*** | 1.67*** | 1.33*** | 0 | 0.7*** | 1*** | 0 | 3*** | 5.19*** | 0 |
| ***bailloni*** | South  Reunion | 2.25*** | 1.98*** | 1.52*** | 0 | 1.12*** | 1.76*** | 0 | 0 | 3.78*** | 3.79*** |
| ***nicolae*** | Seychelles | 2.27*** | 1.38*** | 1.16*** | 0 | 0*** | 1.04*** | -0.19*** | 0.13*** | 0.88*** | 2.04*** |

| c. | Allencay | Longcay | Martinique | St  Barthélémy | Raso | Cima | Mclara | Vila | Selvagem | Funchal | North  Reunion | South  Reunion | Seychelles | Mean | s.d. |
| --- | --- | --- | --- | --- | --- | --- | --- | --- | --- | --- | --- | --- | --- | --- | --- |
| Tajima's D test |  |  |  |  |  |  |  |  |  |  |  |  |  |  |  |
| Sample size | 19 | 20 | 44 | 8 | 18 | 18 | 15 | 19 | 10 | 4 | 28 | 32 | 41 | 21 | 12 |
| S | 17 | 14 | 1 | 48 | 61 | 64 | 49 | 49 | 37 | 24 | 14 | 47 | 14 | 34 | 21 |
| Pi | 2053 | 1930 | 2302 | 1901 | 2279 | 1349 | 1914 | 1963 | 2845 | 2051 | 2495 | 2413 | 2543 | 2157 | 379 |
| Tajima's D | -0.47 | -0.27 | 7.39 | -2.59 | -2.23 | -0.61 | -0.95 | -1.35 | -2.40 | -1.12 | -1.18 | -2.40 | 0.34 | -0.60 | 2.57 |
| p-value | 0.352 | 0.419 | 1 | 0 | 0 | 0.285 | 0.193 | 0.071 | 0 | 0 | 0.104 | 0.002 | 0.718 | 0.24185 | 0.31477 |
| Fu's FS test |  |  |  |  |  |  |  |  |  |  |  |  |  |  |  |
| No. of alleles | 19 | 20 | 44 | 8 | 18 | 18 | 15 | 19 | 10 | 4 | 28 | 32 | 41 | 21 | 12 |
| Theta_pi | 2053 | 1930 | 2302 | 1901 | 2279 | 1349 | 1914 | 1963 | 2845 | 2051 | 2495 | 2413 | 2543 | 2157 | 379 |
| Exp. no. of alleles | 19 | 20 | 44 | 8 | 18 | 18 | 15 | 19 | 10 | 4 | 28 | 32 | 41 | 21 | 12 |
| FS | 2.45 | 2.27 | 0.68 | 4.21 | 2.67 | 2.12 | 2.88 | 2.40 | 4.14 | 5.83 | 1.81 | 1.48 | 0.97 | 2.61 | 1.42 |
| p-value | 0.624 | 0.602 | 0.537 | 0.731 | 0.722 | 0.533 | 0.599 | 0.624 | 0.834 | 0.608 | 0.752 | 0.667 | 0.658 | 0.65315 | 0.08694 |

| d. | Allencay | Longcay | Martinique | St  Barthélémy | Raso | Cima | Mclara | Vila | Selvagem | Funchal | North  Reunion | South  Reunion | Seychelles | Mean | s.d. |
| --- | --- | --- | --- | --- | --- | --- | --- | --- | --- | --- | --- | --- | --- | --- | --- |
| Tajima's D test |  |  |  |  |  |  |  |  |  |  |  |  |  |  |  |
| Sample size | 19 | 20 | 41 | 5 | 18 | 18 | 14 | 18 | 10 | 4 | 18 | 33 | 41 | 19.92 | 11.85 |
| S | 4 | 4 | 1 | 6 | 20 | 11 | 13 | 24 | 4 | 2 | 11 | 1 | 14 | 8.85 | 7.39 |
| Pi | 1434 | 1348 | 1629 | 916 | 1515 | 891 | 1042 | 1348 | 1940 | 1124 | 1753 | 1723 | 1803 | 1420 | 347 |
| Tajima's D | 0.14 | -0.45 | 1.58 | -2.82 | -3.04 | 0.78 | -1.57 | -2.84 | 0.02 | 0.59 | -2.84 | 9.49 | -1.35 | -0.18 | 3.3 |
| p-value | 0.62 | 0.35 | 0.95 | 0 | 0 | 0.81 | 0.06 | 0 | 0.55 | 0.84 | 0 | 1 | 0.09 | 0.4 | 0.4 |
| Fu's FS test |  |  |  |  |  |  |  |  |  |  |  |  |  |  |  |
| No. of alleles | 19 | 20 | 41 | 5 | 18 | 18 | 14 | 18 | 10 | 4 | 18 | 33 | 41 | 19.92 | 11.85 |
| Theta_pi | 1434 | 1348 | 1629 | 916 | 1515 | 891 | 1042 | 1348 | 1940 | 1124 | 1753 | 1723 | 1803 | 1420 | 347 |
| Exp. no. of alleles | 18.88 | 19.86 | 40.5 | 4.99 | 17.9 | 17.83 | 13.91 | 17.89 | 9.98 | 3.99 | 17.91 | 32.7 | 40.55 | 19.76 | 11.7 |
| FS | 20.67 | 19 | 4 | 4.51 | 5.42 | 4.41 | 2.4 | 12.47 | 3.75 | 5.23 | 4 | 11.4 | 21 | 6 | 4 |
| p-value | 1 | 1 | 1 | 0.59 | 0.97 | 0.92 | 0.56 | 1 | 0.8 | 0.56 | 1 | 1 | 1 | 0.88 | 0.18 |

Supplementary Material 8: Gene trees obtained by MrBayes and *BEAST

Gene trees obtained by MrBayes for a. All mitochondrial markers, b. All nuclear markers, c. All markers.

Blue: *lherminieri*, green: *boydi*, red: *baroli*, grey: *bailloni*, black: *nicolae*, yellow: *dichrous*

d. *BEAST gene tree for mitochondrial markers


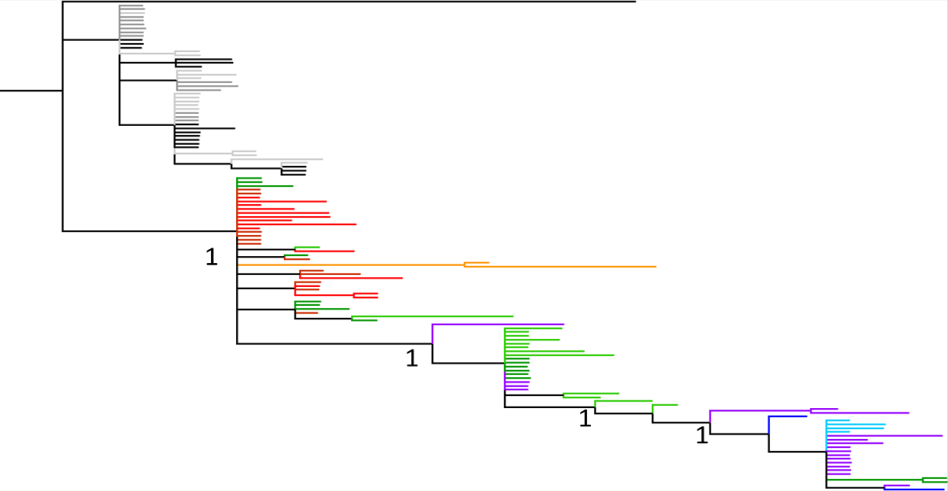


b.

*
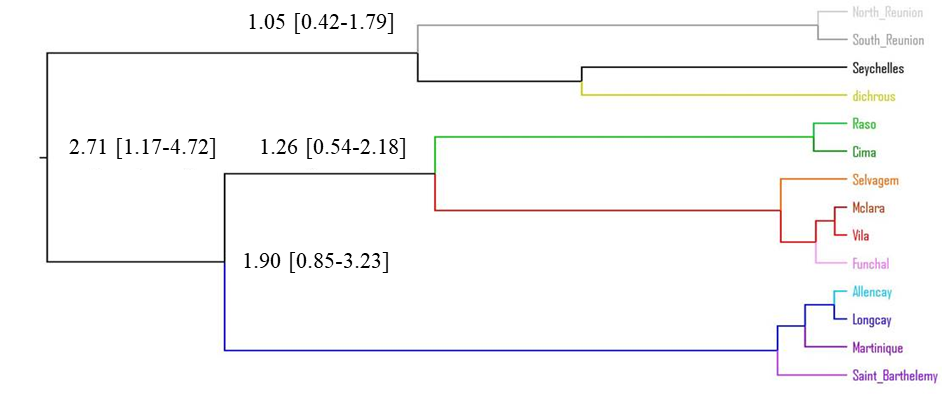
*
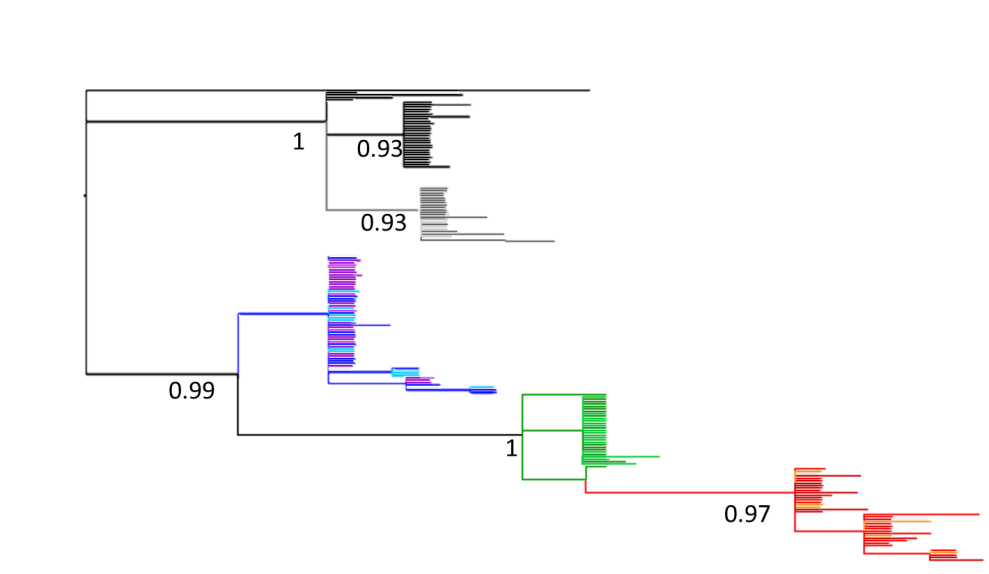


1

1

1

1

0,99

0,93

0,98

0,90


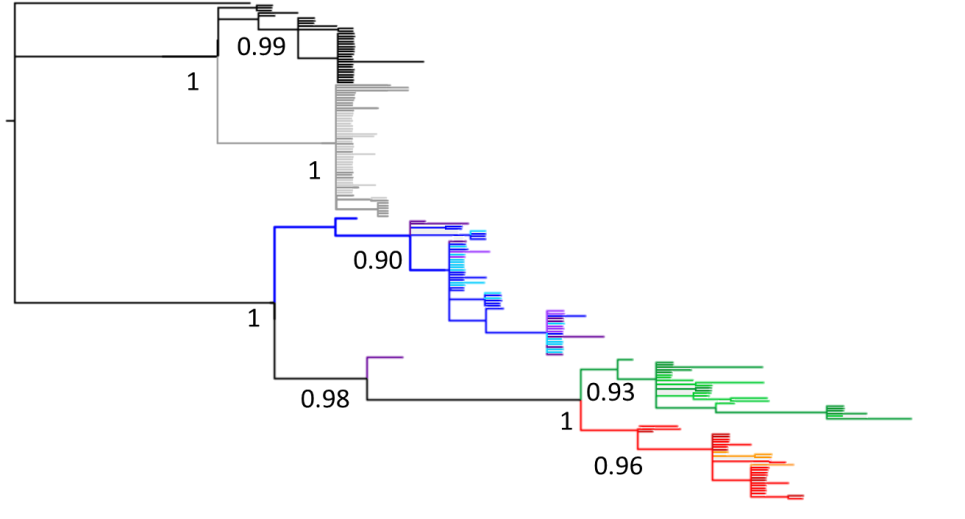


a.

d.

c.

Neighbor networks on e. *cox1*, f. *cytb*, g. the Control Region, h. *pax*, i. *csde*, j. *tpm*, k. *irf2*, l. *βfib*, m. *rag1*. The scale bars show how the length of a branch translates in sequence divergence. The unit is divergent nucleotides divided by the length of the sequence analysed.


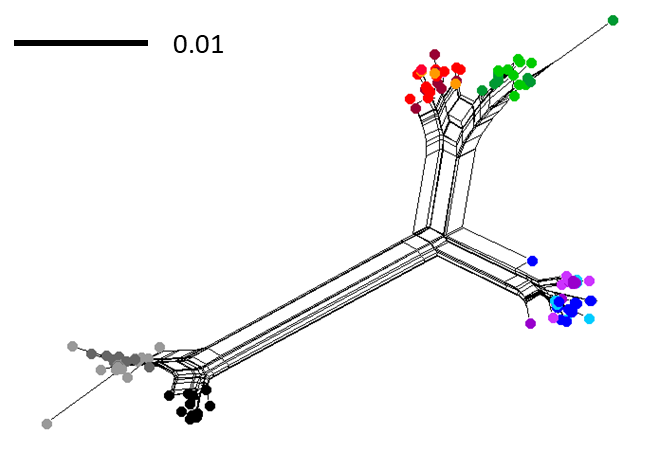


f. *cytb*

g. CR


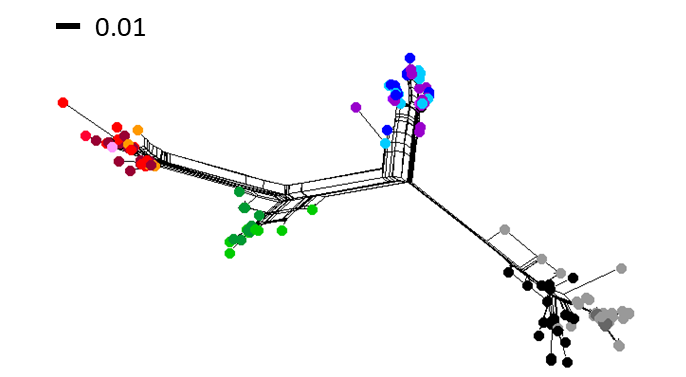


e. *cox1*


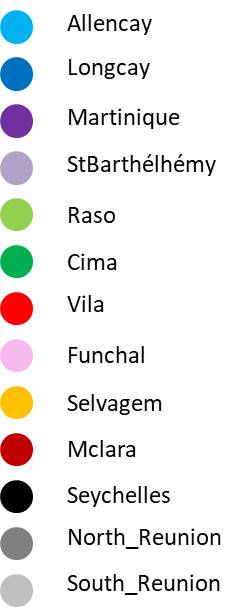
*
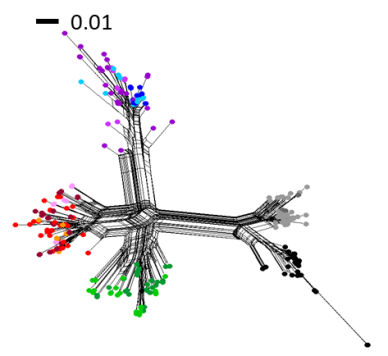
*


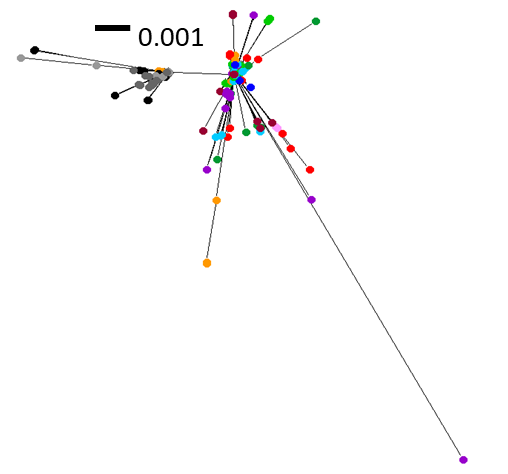


i. *csde*


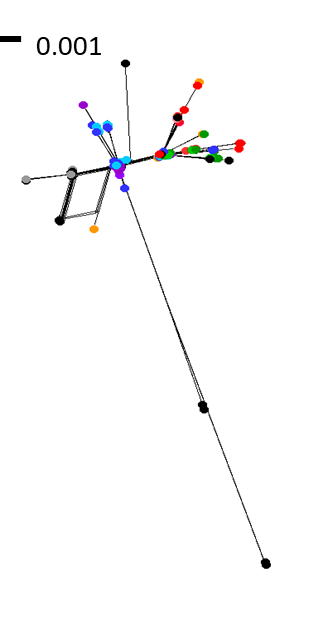


h. *pax* Pax


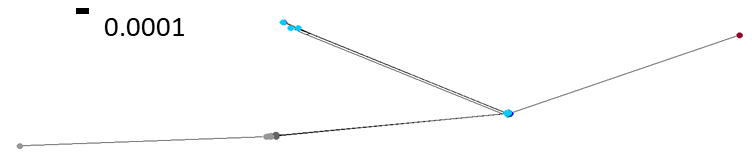


j. *tpm*


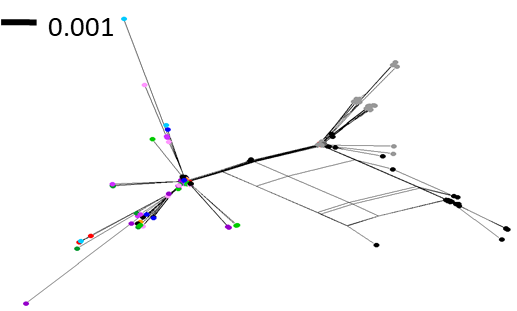


k. *irf2*

*
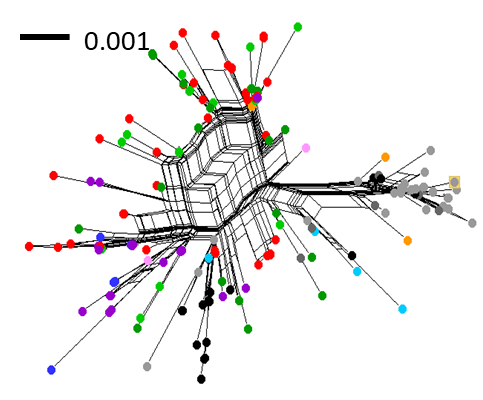
*

m. *rag1*


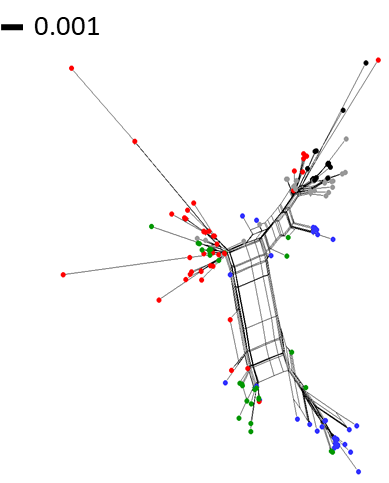


l. *βfib*

Supplementary Material 9: Discordant mito-nuclear data

Assignment to an ocean basin based on nuclear and mitochondrial data were discordant for 33 individuals, for at least one nuclear locus. All of these individuals showed the mitochondrial signature expected based on their geographical sampling location. The haplotype networks of the two haplotypic phases of each nuclear marker showed us than one or two phases of these individuals could be discordant for one to three markers. The fact that two haplotypic phases of one individual could be discordant does not allow us to exclude contamination during lab work as a cause of this pattern. However recent hybridization as well as retention of ancestral polymorphism can also cause these patterns. For one individual, one haplotypic phase is found discordant for the more discriminant nuclear markers but not for mitochondrial markers, which could be indicative of first-generation hybridization. For other individuals, the discordant haplotypic phase at one or several markers could be a remaining of ancestral form of the loci. Further analyses are required to investigate this pattern more in detail, including the sequencing of more loci and correspondance and assignment analyses.

Inventory of discordant individuals in nuclear markers. For each population individuals presenting one or two haplotypic phases that was grouped with the individuals of the other ocean for at least one nuclear marker are indicated here. For each nuclear marker, an “x” indicates that the two haplotypic phases of the individual are discordant with its sampling location, a “v” indicates that only one phase is discordant. The sex is indicated if known.

| Lineage | Population | Individual | *pax* | *csde* | *tpm* | *irf2* | *βfib* | Sex | Geographic placement of dicordant phase |
| --- | --- | --- | --- | --- | --- | --- | --- | --- | --- |
| *lherminieri* | Allencay | Allencay 18 |  |  |  |  | x | M | Indian populations |
| *lherminieri* | Allencay | Allencay 19 |  |  |  |  | x | M | Indian populations |
| *lherminieri* | Longcay | Longcay19 |  |  |  |  | v | M | Indian populations |
| *lherminieri* | Longcay | Longcay2 |  |  |  |  | v | F | Indian populations |
| *lherminieri* | Martinique | BU83 |  |  |  |  | x | M | Indian populations |
|  |  |  |  |  |  |  |  |  |  |
| *boydi* | Raso | 5500040 |  |  |  |  | v | M | Indian populations |
| *boydi* | Cima | 5500491 |  |  |  |  | v | M | Indian populations |
|  |  |  |  |  |  |  |  |  |  |
| *baroli* | Vila | I008058 | v |  |  | v | v | M | Indian populations |
| *baroli* | Vila | I008072 |  |  | v | v | x | M | Indian populations |
| *baroli* | Vila | I008098 |  |  |  | v |  | F | Indian populations |
| *baroli* | Vila | I008099 |  |  |  |  | x | M | Indian populations |
| *baroli* | Selvagem | Selvagem 1 |  |  |  | v | x | F | Indian populations |
| *baroli* | Selvagem | Selvagem 2 |  |  |  |  | x | F | Indian populations |
| *baroli* | Selvagem | SelvagemX1 |  | v |  |  |  | NA | Indian populations |
| *baroli* | Selvagem | SelvagemX2 | v |  |  |  |  | NA | Indian populations |
|  |  |  |  |  |  |  |  |  |  |
| *bailloni* | North Reunion | 142169 |  |  | v | v |  | M | Atlantic populations |
| *bailloni* | North Reunion | 142213 | v |  |  |  |  | F | East Atlantic populations |
| *bailloni* | North Reunion | BY13 |  |  |  | v | x | F | East Atlantic populations |
| *bailloni* | North Reunion | BY15 |  |  | x |  |  | F | Atlantic populations |
| *bailloni* | North Reunion | BY17 | v |  |  |  | v | M | East Atlantic populations |
| *bailloni* | North Reunion | BY22 |  |  | x | v |  | M | Atlantic populations |
| *bailloni* | North Reunion | BY23 | v |  |  | v | x | F | East Atlantic populations |
| *bailloni* | North Reunion | BY25 |  |  | x |  |  | F | Atlantic populations |
| *bailloni* | North Reunion | BY27 |  |  | x |  |  | M | Atlantic populations |
| *bailloni* | South Reunion | 1587 | x |  |  |  |  | F | East Atlantic populations |
| *bailloni* | South Reunion | 142175 |  |  | v |  |  | F | Atlantic populations |
| *bailloni* | South Reunion | 142529 |  |  | x |  |  | F | Atlantic populations |
|  |  |  |  |  |  |  |  |  |  |
| *nicolae* | Seychelles | BW44 | v |  |  |  |  | NA | West Atlantic populations |
| *nicolae* | Seychelles | BW66 | v |  |  |  |  | F | West Atlantic populations |
| *nicolae* | Seychelles | BW68 | v |  |  |  |  | M | West Atlantic populations |
| *nicolae* | Seychelles | GE50909 | x |  |  |  |  | NA | West Atlantic populations |
| *nicolae* | Seychelles | GE50910 | x |  |  |  |  | NA | East Atlantic populations |
| *nicolae* | Seychelles | GE50921 | v |  |  |  |  | NA | West Atlantic populations |

Supplementary material 10 : Bayesian skyline analyses

b.

a.

The four rows correspond to the four sets of clock rates used for *cox1* and *cytb*. The first one is the set used in our main analyses: 0.01588 ± 0.00115 per site per million year for *cox1* (Pereira and Baker 2006) and 0.0189 ± 0.0035 per site per million year for *cytb* (Weir and Schluter 2008). The second uses both rates of Pacheco et al. (2011), 0.00184 for *cox1* and 0.00178 for *cytb*. Population size is displayed as θ. Ne is obtained by multiplying it by the mean substitution rate.

a. *lherminieri* b*. boydi* c. *baroli* d.*bailloni* e. *nicolae*


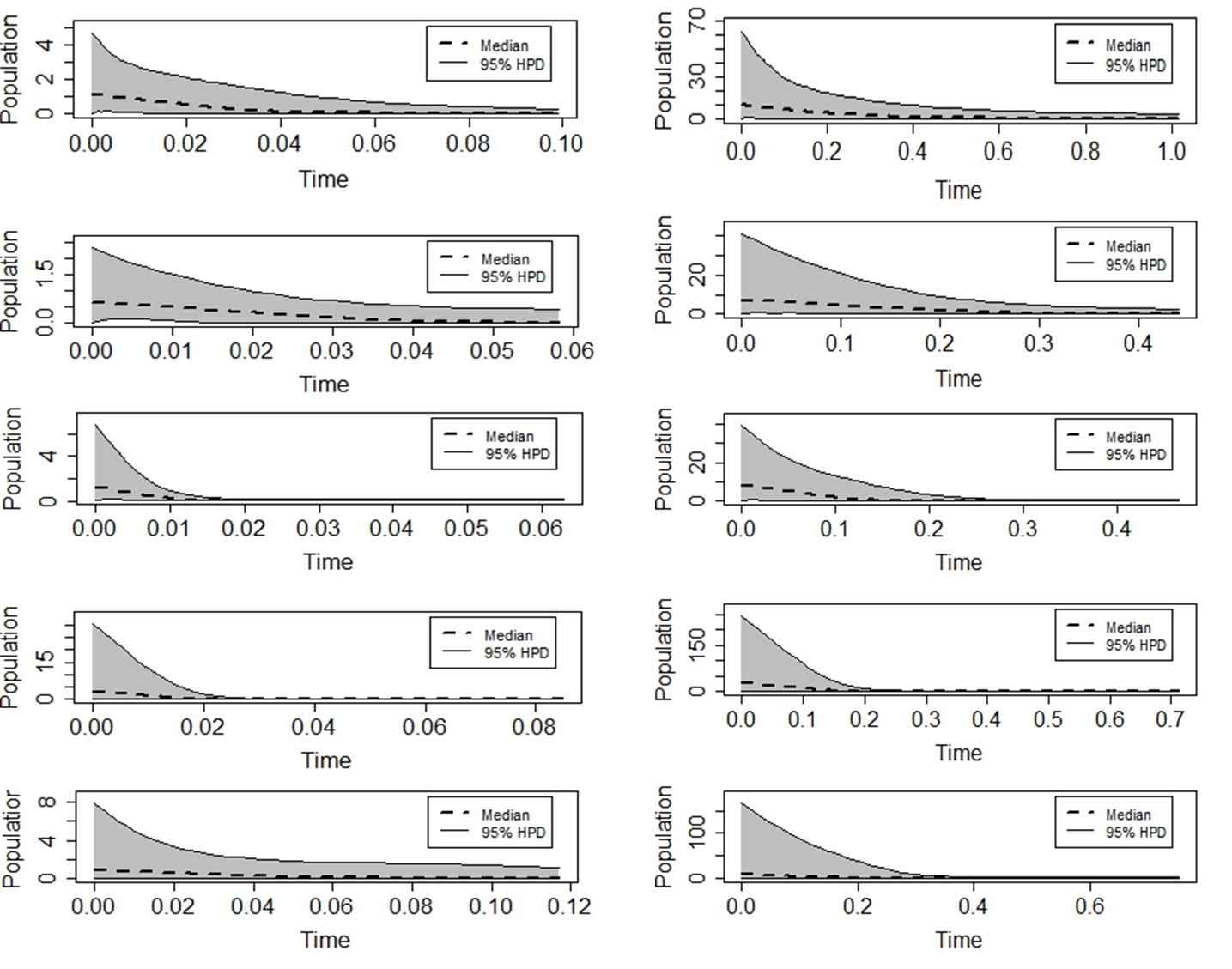


e.

d.

c.

b.

a.
